# A Comparison Analysis of Quality and Metabolic Compounds in Lilies with Different Drying Treatments

**DOI:** 10.3390/foods13142206

**Published:** 2024-07-13

**Authors:** Lixia Xie, Jiajia Liu, Haoyu Wu, Yueyan Zhong, Xueying Liu, Guangli Li, Zhi Liu

**Affiliations:** 1 Hunan Provincial Engineering Research Center of Lily Germplasm Resource Innovation and Deep Processing, Hunan University of Technology, Zhuzhou 412007, China; xielx@hut.edu.cn (L.X.); 17735270742@163.com (J.L.); 15719238982@163.com (H.W.); 18390611596@163.com (Y.Z.); liuxueying@hut.edu.cn (X.L.); 2 Hunan Key Laboratory of Biomedical Nanomaterials and Devices, College of Life Sciences and Chemistry, Hunan University of Technology, Zhuzhou 412007, China; 3 College of Agriculture and Biotechnology, Hunan University of Humanities, Science and Technology, Loudi 417099, China

**Keywords:** lily, drying treatments, quality, metabolomics, HCA

## Abstract

The present study aimed to investigate the variations in the nutritional composition, antioxidant capacity, and metabolite profile of lilies subjected to different drying treatments, including vacuum freeze drying (VFD), hot air drying (HAD), vacuum drying (VD), and infrared drying (ID). The results show that VFD provided better preservation of the original coloration and displayed the highest levels of total amino acid content, total phenolic content, total flavonoid content, and polysaccharide and alkaloid content. Our results reveal that VFD treatment can be employed to obtain high-quality lilies with desirable appearance characteristics and nutrient compositions. Metabolomics analysis identified a total of 464 metabolites from various dried lilies. Differential metabolite screening found 150 differential metabolites across all pairwise comparisons. Hierarchical clustering analysis (HCA) indicated that lilies subjected to VFD treatment exhibited a higher abundance of steroids, saponin, flavonoids, and phenolic glycoside, whereas those subjected to HAD, VD, or ID treatments showed relatively elevated levels of specific amino acids or derivatives. This study elucidates the significant impact of various drying treatments on the quality and metabolic profile of lilies, thereby providing valuable insights for enhancing the nutritional quality of processed lilies.

## 1. Introduction

The genus *Lilium*, a perennial bulbous herb of the Liliaceae family, is a significant wild resource, with approximately 55 species in China, 21 species in North America, and 10 species in Europe and the Caucasus [1]. The bulbs of Lilium species (or lily bulbs) consist of fleshy scales that provide essential nutrients for their growth. They are rich in amino acids, nucleosides, vitamins, trace elements, dietary fiber, phospholipids, and other nutrients. For instance, the starch content accounts for about 11–22% of the dried weight of the bulb, and the free amino acid content ranges from 0.54 to 4.96% [2]. In addition to their nutritional value, lily bulbs contain various important compounds, such as alkaloids, steroidal saponins, flavonoids and polysaccharides [3,4], which play crucial roles in plants’ protection against biotic and abiotic stresses. Specifically, lily plants have been identified as an exceptionally abundant source of steroidal saponins, with levels reported as high as 13.184 mg/g in *L. longiflorum* bulbs [2]. These compounds exhibit a wide range of biological activities, including antibacterial, anti-inflammatory, antitumor, antioxidant, and proliferation inhibition properties [5,6,7,8].

Fresh lily bulbs have a moisture content exceeding 70%, which renders them highly susceptible to rot during storage and results in a shorter shelf life and significant economic losses. Additionally, inherent enzymes make fresh lily bulbs prone to browning, leading to dark color formation and negatively impacting product quality [9,10]. To extend the shelf life and maintain nutritional value for better commercialization, immediate processing methods that reduce moisture content must be employed after harvest. Drying is one of the most effective solutions for this issue as it removes water from agricultural products and reduces water activity. However, drying processes can lead to the degradation of nutrients and metabolites, resulting in the deterioration of product quality such as changes in color, flavor, microbial load, texture, taste, aroma, or nutritional properties. Therefore, there has been a significant surge in interest regarding the impact of drying treatments on agricultural products in recent years [11,12,13]. Several studies have focused on examining the effects of various drying treatments on the microstructure, nutrients, antioxidant activities, bioactive components, and volatile profiles of lily bulbs [14,15,16,17]. Physical properties, along with other quality attributes including total phenolic content (TPC), and diphenyl-1-picrylhydrazyl (DPPH) and ferric reducing antioxidant potential (FRAP) assays are usually employed to evaluate the efficacy of different drying treatments in the processing of agricultural products. However, the limited physicochemical properties only offer preliminary insights into specific drying treatments. Further research is warranted to investigate the chemical composition characteristics of agricultural products under different drying treatments and their correlation with qualitative indicators. Therefore, comprehensive studies on the metabolites exhibiting significant differences among various drying methods are imperative.

Metabolomics provides a powerful approach for investigating comprehensive changes in experimental groups, using advanced analytical technologies such as nuclear magnetic resonance (NMR) and liquid or gas chromatography coupled with mass spectrometry (LC or GC/MS), in conjunction with multivariate statistical analysis [18,19]. The potential of metabolomics has already been acknowledged in several studies investigating the impact of various drying treatments on plant-derived products [20,21]. Metabolomics enables the comprehensive identification of a diverse array of molecules that may undergo alterations due to drying processes. Li et al. examined the variations in *Lonicerae japonicae* flos processed with different drying techniques through chemometrics combined with GC-MS and UHPLC-HRMS analysis [20]. Polat et al. investigated the impact of various drying methods on the phenolic compounds of black carrot pomace using targeted metabolomics based on HPLC-DAD-ESI-MS/MS [21]. A previous study reported the comparison of different lily bulbs using a UPLC-ESI-MS/MS-based untargeted metabolomics approach, wherein steroid saponins were identified as the key bioactive compounds in medicinal lilies [18]. Additionally, targeted UHPLC-MS analysis revealed Regaloside A as the characteristic component in *L. lancifolium* bulbs when investigating the effects of various drying processes on its representative chemical components [16]. Therefore, limited research exists regarding the effect of drying treatments on lily bulbs.

Irrespective of previous findings on the quality attributes of lily bulbs, it is imperative to conduct a systematic and comprehensive investigation into the impact of different drying techniques on the chemical constituents and other characteristics of lily bulbs. In this study, a metabolomics approach combining liquid chromatography with tandem mass spectrometry (LC-MS/MS) was employed to elucidate the metabolite profiles of lilies during various drying procedures, namely vacuum freeze drying (VFD), hot air drying (HAD), vacuum drying (VD), and infrared drying (ID). This research is of great significance for selecting an optimal drying technique that can yield exceptionally high-quality dried lily products.

## 2. Materials and Methods

### 2.1. Chemicals

All chemicals utilized in this study were of analytical grade. Analytical-grade standards of 18 amino acid were obtained from Merck (Merck, Darmstadt, Germany). LC-MS-grade acetonitrile and methanol were purchased from Merck (Merck, Darmstadt, Germany); LC-MS-grade formic acid was purchased from Xiya Chemical Technology Co., Ltd. (Linxi, China). Other chemicals such as gallic acid, rutin, colchicine, Folin–Ciocalteu, Trolox (6-hydroxy-2,5,7,8,-tetramethylchromane-2-carboxylic acid) and TPTZ (2,4,6-tri(2-pyridyl)-S-triazine) were purchased from Shanghai Aladdin Biochemical Technology Co., Ltd. (Shanghai, China). Deionized water was purified using a Millipore Milli-Q® Direct 16 water purification system (Millipore, Billerica, MA, USA).

### 2.2. Preparation and Drying Treatments

The lily bulbs were obtained from a company situated in Longhui city, Hunan province, which is renowned as one of the prominent regions for cultivating lilies (*L. brownie* var. *viridulum*) in China. The raw materials selected for this study were high-quality fresh lily slices, characterized by a smooth and delicate texture, uniform scale size, and intact appearance without any blemishes or insect damage. The initial moisture content of the lily slices was about 65%. The lily slices underwent blanching treatment, a commonly employed commercial method for the production of lily products. The blanched lily slices were divided into four groups, with each group consisting of three replicates, resulting in a total of 12 samples. The samples from each group were subjected to different drying treatments (VFD: vacuum freeze drying; HAD: hot air drying; VD: vacuum drying; and ID: infrared drying) in the Engineering Research Center of Lily Germplasm Resource Innovation and Deep Processing located in Zhuzhou, China.

VFD: The lilies were first frozen at −80 °C for 2 h. The frozen materials were arranged uniformly on a tray, and then put into the chamber of a vacuum lyophilizer (LGJ-12SN, Beijing Songyuan Huaxing Technology Development Company, Ltd., Beijing, China). The heating shelf temperature was set to 60 °C. The pressure of the drying chamber during the drying process was maintained at 50 Pa, while the cold trap temperature was controlled at −40 °C. The samples were dehydrated for 15 h.

HAD: The lilies were spread uniformly on a stainless steel tray and placed in an air drying oven (101-2AB, Tianjin Taisite Instrument Company, Ltd., Tianjin, China). The temperature was controlled and held at 65 °C for 18 h. 

VD: The lilies were evenly distributed on a stainless steel tray and dried in a vacuum chamber (DZ-1BCIV, Tianjin Taisite Instrument Company, Ltd., Tianjin, China) under vacuum conditions of −80 kPa and a temperature of 65 °C for 18 h.

ID: The lilies were subjected to infrared drying in an infrared-ray oven (WS70-1, Hangzhou Qiwei Instrument Company, Ltd., Hangzhou, China). The drying was performed at 100 °C with a power consumption of 1200 W for 1 h. 

After the drying treatments, the moisture content of samples was determined to be below 9.0 ± 1.0 g/100 g dry weight (DW). The dried samples were vacuum-packed and stored at −80 °C until the analyses.

### 2.3. Color Measurement

The color parameters of various dried lily samples were determined using a color measurement device (SR211, Shenzhen Weifu Photoelectric Technology Company, Ltd., Shenzhou, China) with the CIE Lab color scale (L*, a*, b*). Five replicated measures were made from different parts of the given samples. Then, the average values were calculated. The total color change (Δ*E*) for each sample was computed using the formula given below.

$$∆E=\sqrt{\left[{\left(L_{p}^{*}-L_{dp}^{*}\right)}^{2}+{\left(a_{p}^{*}-a_{dp}^{*}\right)}^{2}+{\left(b_{p}^{*}-b_{dp}^{*}\right)}^{2}\right]}$$

where the color brightness is represented by the parameter L*; the attribute a* indicates green colors with negative values and red colors with positive values; and the attribute b* represents blue colors with negative numbers and yellow colors with positive numbers. p refers to the fresh lily sample, while dp specifically denotes the dried lily sample.

### 2.4. Amino Acid Analysis

In the present work, the content of total amino acids, including aspartic acid, glutamic acid, serine, glycine, histidine, arginine, threonine, alanine, proline, tyrosine, valine, methionine, cystine, isoleucine, leucine, phenylalanine, and lysine, was measured as follows. The lily samples were ground into powder, and subsequently 1.00 g of the powdered material was dissolved in water, followed by ultrasonic extraction at room temperature for a duration of 30 min. After centrifugation for 10 min, the supernatant was collected. The extraction step was repeated three times, and the resulting supernatants were collected to achieve a final volume of 50 mL. The amino acid concentration in the extraction solution was diluted using 0.2 mol/L sodium citrate buffer (pH 2.2). The pH-adjusted solution was filtered through a Millipore nylon membrane filter with a pore size of 0.22 μm. Amino acid quantification was performed using a Biochrom 20 automatic amino acid analyzer (Biochrom Ltd., Holliston, MA, USA). Individual amino acids were identified using spiking with known amino acids. The concentration was expressed as mg/g dry weight. 

### 2.5. Determination of Total Phenolic Content

The determination of total phenolic content (TPC) was conducted as follows. The lily powder was taken, with each sample accurately weighing 1.00 g, and placed into a 50 mL centrifuge tube. Subsequently, a methanol/water mixture (50:50) with a volume of 40 mL was added along with hydrochloric acid to adjust the pH to 2.0. Ultrasonic extraction was performed at a temperature of 30 °C for a duration of 30 min, followed by centrifugation at 15 °C for 10 min at a speed of 2500 r/min. The resulting supernatant was collected, while the residue underwent repeated extraction according to the aforementioned procedure. The determination of TPC was carried out using the Folin–Ciocalteu method as described in [22]. A 1.00 mL aliquot of diluted phenolic extract (1:10 *v*/*v* in deionized water) was mixed well with 5.00 mL of a 0.20 mol/L Folin–Ciocalteu reagent, thoroughly shaken, and allowed to stand for 3 min. Subsequently, 4.00 mL of a 7.5% (*w*/*v*) sodium carbonate solution was added, followed by another round of vigorous shaking. The reaction mixture was kept at room temperature in the absence of light for 60 min. Finally, the absorbance at 765 nm was measured by a UV-spectrophotometer (UV-1800, Shanghai Mapada Instrument Company, Ltd., Shanghai, China). A sample prepared with deionized water instead of the diluted extracts served as a control. To establish a standard curve, gallic acid solutions with concentrations ranging from 0 to 10.00 mg/L were employed. The values were expressed as mg gallic acid equivalent (GAE)/g dry weight.

### 2.6. Determination of Total Flavonoids Content

The total flavonoid content (TFC) was determined through the following procedural steps. The lily powder was prepared, with each sample weighing 1.00 g, and was dissolved in 50 mL of methanol. The mixture underwent ultrasonic extraction at a speed of 2500 r/min for 30 min followed by filtration to collect the filter solution. The residue was subjected to repeated extraction using the aforementioned step. The total flavonoid content was assessed according to the modified method [23]. Briefly, 1.00 mL of extraction solution was added into tubes containing 2.00 mL of distilled water and 1.00 mL of 5% NaNO_2_. After 6 min, 1.00 mL of 10% Al(NO_3_)_3_ solution was introduced, followed by an additional incubation for another 6 min. Subsequently, 5.00 mL of 4% NaOH was added to the mixture and allowed to stand for a duration of 15 min before measuring the absorbance at a wavelength of 510 nm using a spectrophotometer. A control sample was prepared with deionized water instead of the diluted extracts. Rutin with a concentration ranging from 0 mg/mL to 0.10 mg/mL was used as the standard sample, and the values were expressed as mg rutin equivalent (RE)/g dry weight.

### 2.7. Determination of the Antioxidant Activity by FRAP Assay

The ferric reducing antioxidant potential (FRAP) assay was conducted following the protocol described by [24]. The FRAP reagent was produced by combining 2.50 mL of a 10.00 mM TPTZ solution in 40 mM HCl, 2.50 mL of a 20.00 mM ferric chloride hexahydrate in deionized water and 25 mL of a 0.30 mM acetate buffer (pH 3.6). After incubated at 37 °C for 30 min, a 1.80 mL aliquot of freshly prepared FRAP reagent was mixed with 180.00 μL of deionized water and 20.00 μL of the diluted phenolic extracted or standard or reagent blank. The mixture was allowed to stand for 30 min at 37 °C, and finally the absorbance at 595 nm was recorded. Calibration was performed using Trolox^®^ dissolved in methanol within the concentration ranging from 0 to 100.00 mg/L. The reducing power was expressed as mg Trolox^®^/g dry weight.

### 2.8. Determination of Polysaccharide

A total of 1.00 g of lily powder was dissolved in 100.00 mL of deionized water and subjected to extraction at 100 °C for 2 h. The mixture was then cooled to room temperature and adjusted to the desired volume with deionized water. Subsequently, centrifugation was performed at a speed of 6000 r/min for 10 min, followed by filtration of the supernatant. Then, 15 mL of ethanol was added to the filtrate, thoroughly mixed and shaken, and subsequently placed in a refrigerator at 4 °C for 3 h. After a centrifugation at 6000 r/min for 10 min, the supernatant was discarded and the precipitate was washed repeatedly with ethanol. Finally, the resulting polysaccharide extract was dissolved in heated water until a constant volume of 10.00 mL was achieved. The polysaccharide content was quantified using the phenol–sulfuric acid method [25]. 

### 2.9. Determination of Alkaloid

A total of 1.00 g of lily powder was combined with 50 mL of acetoacetate sodium acetate buffer (pH 3.6), followed by reflux heating for 60 min, subsequent centrifugation, and collection of the resulting supernatant to obtain the alkaloid extract. A 3.00 mL aliquot of alkaloid extract was combined with 5.00 mL of bromocresol green, followed by the addition of 10.00 mL of dichloromethane. The mixture was vigorously shaken for 3 min and allowed to stand for 30 min. Subsequently, the dichloromethane layer was treated with 0.50 g of anhydrous sodium sulfate and left undisturbed for another 30 min. The absorbance at a wavelength of 414 nm was measured to determine the alkaloid content using colchicine as the standard substance.

### 2.10. LC-MS/MS Analysis 

The extraction of the metabolic components was firstly conducted. A 100.00 mg portion of lily powder was weighed into a 2 mL centrifuge tube, following by the addition of 1.00 mL methanol–water (*v*/*v* 70:30) and 3 mm steel balls. The mixture was ground using a JXFSTPRP-48 fully automated high-speed sample grinder (Shanghai Jingxin Industrial Development Co., Ltd., Shanghai, China) for 3 min at 70 Hz, followed by sonication at a low temperature for 10 min with a frequency of 40 KHz. After centrifugation at 12,000 r/min and 4 °C for 10 min to remove residues, the upper layer was taken and diluted to various ratios ranging from 2 to 100. Then, 0.10 mg/L lidocaine was added into the supernatant as an internal standard. Finally, the sample was filtered through a PTFE membrane filter with a pore size of 0.22 μm and analyzed using an UHPLC-MS. A quality control (QC) sample was prepared by equally pooling extracts from all samples to assess the repeatability and stability of the method.

Briefly, 2.00 μL samples were injected into an Ultimate 3000 liquid chromatograph. During the analysis, two QC samples were inserted into each test group sample. The mobile phase consisted of acidified water (0.1% formic acid) (A) and acetonitrile (B). Separation was performed on a C18 column (Zorbax Eclipse, Agilent technologies, 1.8 μm, 2.1 mm × 100 mm) at 30 °C and a flow rate of 0.3 mL/min using the following gradient: 95:5 Phase A/Phase B at 0–2 min; 70:30 Phase A/Phase B at 6–7 min; 22:78 Phase A/Phase B at 12–14 min; 5:95 Phase A/Phase B at 17–20 min; 95:5 Phase A/Phase B at 21–25 min. The effluent was connected to a Q-Exactive HF Mass system (Thermo Fisher Scientific, Waltham, MA, USA).

The optimized parameters were as follows: ion source, HESI; capillary temperature, 330 °C; spray voltage, 3.50 kV; AGC target, 1e6; the flow rate of auxiliary gas, 12.0 arbs; the flow rate of sheath gas, 50.0 arbs; scanning range, 100~1500 m/z; scanning speed, 1000 Da/s; and dynamic exclusion, 4.5 s. The resolution for the MS1 and MS2 was 120,000 and 60,000, respectively; collision energy, 12.5 eV, 25 eV and 35 eV; maximum IT, 30 ms; and isolation window, 3. The Q Exactive HF was operated in both positive and negative ion modes.

The raw LC/MS data were analyzed using Compound Discover 3.2 software, which included retention time correction, peak recognition, peak extraction, peak integration and peak alignment. The identification of metabolites was carried out by searching through our internal database and public databases, such as Thermo mzCloud, ThermomzValut and ChemSpider. The relative metabolite contents were expressed as chromatographic peak area integrals. 

### 2.11. Statistical Analysis

Triplicate analyses were conducted for each sample, and the results were presented as means ± SD. Statistically significant differences were analyzed using SPSS statistics (Version 26.0) software. Principal component analysis (PCA) was carried out using the factoextra 1.0.7 package in RStudio 4.3.1. For sample classification and discrimination, orthogonal projection to latent structures-discriminant analysis (OPLS-DA) was employed with SIMCA-P 4.1 software. Significant differential metabolites were selected based on the variable weight value (VIP) obtained by the OPLS-DA model, Student’s t-test *p*-value, and fold change (FC). Those with a *p*-value < 0.05, FC ≥ 2 or ≤0.5 and VIP ≥ 1 were considered as differential metabolites between two groups. Hierarchical cluster analysis (HCA) and Venn diagram analysis were conducted using the ComplexHeatmap 2.18.0 and VennDiagram 1.7.3 packages in RStudio, respectively. In HCA, the signal intensities of each metabolite were normalized.

## 3. Results

### 3.1. Humidity and Drying Time

In the present work, the humidity and drying time are listed in Table 1. It is evident from this table that all drying treatments yielded a humidity level below 10%. The drying time exhibited considerable variability depending on the specific treatment employed. Compared to the other three drying treatments, ID exhibits the shortest drying time due to its higher temperature. Xu et al. dried tangerine peel with different methods and found that the drying time depended on both the drying method and temperature [26]. They also stated that infrared drying was the fastest method to dry tangerine peels among all the methods.

### 3.2. Color

**Figure 1:**
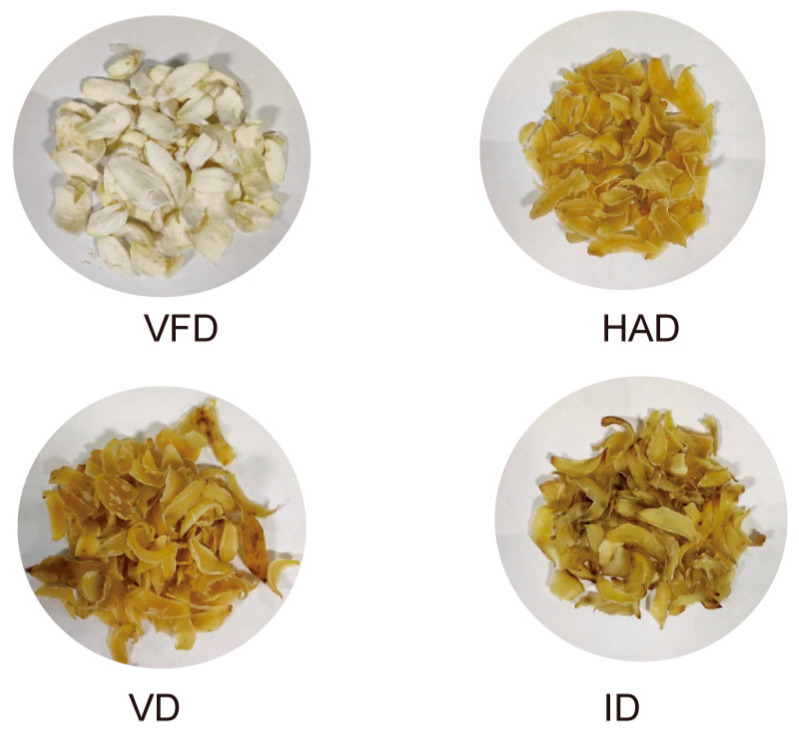
The appearance of different drying-treated lily samples.

The colors of dried lilies from the four different drying treatments are presented in Figure 1. It can be visually observed from the photographs of dried lily samples that significant color changes occurred as a result of the various drying treatments.

The color parameters of dried lilies, with reference to the fresh sample, are summarized in Table 2. The brightness values (L*) of dried lilies ranged from 67.32 to 90.88. The VFD-treated sample exhibited significantly higher L* value compared to those dried using other methods, indicating its brighter appearance, which could be attributed to the lower drying temperatures employed during VFD treatment. The redness values (a*) were determined to be positive for all samples except for the VFD sample, which exhibited a negative greenness value. The ID samples showed relatively high redness values of 4.69 ± 0.6. Additionally, the ID sample displayed a relatively high yellowness value (b*) of 24.39 ± 0.7, while the VFD sample had the lowest b* value of 12.77 ± 1.6. The Δ*E* values ranged from 8.84 to 16.60, serving as an indicator for evaluating color changes after drying treatments. Compared to other drying treatments, the VFD treatment resulted in lower Δ*E* values, suggesting minimal color alteration and better preservation of the original coloration. Infrared drying yielded the poorest color, consistent with previous findings in studying red pepper [27] and pleurotus eryngii [28]. Those changes in the a* and b* parameters indicated an increased presence of red and yellow in the dried lily samples. It can be explained that prolonged exposure to high air temperature led to browning, primarily caused by Maillard reactions, caramelization reactions, and ascorbic acid-induced browning [29]. Browning can significantly impact the quality of lily products, and the color thereof plays a pivotal role in determining their acceptability. Nevertheless, it is noteworthy that while VFD can effectively preserve the color of lilies to a significant extent, it requires higher energy consumption compared with other methods of drying.

### 3.3. Amino Acids Composition

Lilies are renowned for their high amino acid compound content, which is essential for human nutrition. The free amino acid profiles of lilies subjected to various drying treatments were determined as presented in Table 3. These results reveal that arginine was the predominant amino acid with the highest concentration, accounting for 40.8~48.7% of total amino acids (TAA), followed by glutamic acid (20.3~27.3%), leucine (7.1%~11.8%), and aspartic acid (6.4~9.1%). These amino acids have previously been reported to be present in large quantities in lilies, constituting approximately 44.02% of the total amino acid content in fresh lilies [30]. Free amino acids are classified as taste-active compounds and play crucial roles in eliciting characteristic flavors of food. According to sensory attributes, arginine is generally associated with a bitter taste, while glutamic acid and aspartic acid primarily contribute to an umami taste [31]. The high levels of arginine, glutamic acid and aspartic acid observed in lilies agreed well with their known bittersweet taste. 

In this study, no significant differences were found in the composition of amino acids between lily samples subjected to the HAD and VD treatments. Cysteine was not detected in any dried lily samples, consistent with previous reports [30]. In the current study, the levels of arginine and leucine in the HAD and VD samples were lower than those in the VFD and ID samples. The total amino acid (TAA) contents reached 1.7% for the VFD samples, which was significantly higher than those in the HAD and VD samples. High temperature is conducive to the occurrence of the Maillard reaction, which reduces the amino acid content. Several studies reported that heat treatment may change the composition of amino acids [32]. These results suggest that the susceptibility of amino acids to drying treatments can render them prone to potential losses, alterations, or even destruction during the processing procedures.

### 3.4. The Content of Total Phenols and Total Flavonoids

**Figure 2:**
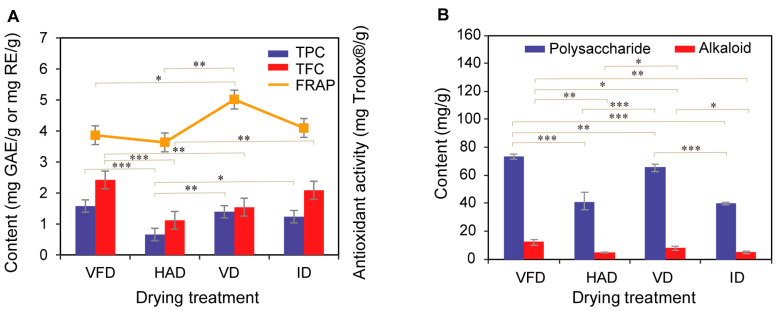
Effect of different drying treatments on lily. (**A**) TPC, TFC and FRAP; (**B**) polysaccharide and alkaloid. * indicates significant difference, * *p* < 0.05, ** *p* < 0.01, and *** *p* < 0.001.

The drying process significantly influenced the content of total phenols and total flavonoids, resulting in remarkable differences. The TPC of dried lilies ranged from 0.66 to 1.58 mg GAE/g dw, while the TFC varied from 1.12 to 2.42 mg RE/g dw. As displayed in Figure 2A, higher TPCs were observed in samples after VFD, whereas lower levels were found in samples after HAD treatment. A similar trend was observed for the TFCs of dried samples, which can be attributed to the susceptibility of flavonoids and phenolics to degradation or oxidation by light, heat, and oxygen during heat-drying treatments on lilies. In contrast, vacuum and low-temperature conditions during the VFD process minimized the decomposition of flavonoids and phenolics compounds. Compared to the HAD, the application of infrared treatment resulted in higher levels of flavonoids and phenolics, consistent with a previous study demonstrating increased total phenols in infrared-dried rice husks [33]. Exposure to infrared radiation activates the release of covalently linked phenol compounds from some agricultural products.

### 3.5. The Antioxidant Capacity

Lilies exhibit remarkable antioxidant capacity, attributed to their abundant presence of antioxidative substances, such as phenolics and flavonoids mentioned above. It is widely acknowledged that the drying process can influence the levels of unstable antioxidants in medicinal materials or agricultural products. Therefore, it is meaningful to evaluate the antioxidant capacity of different drying-treated lilies. In this study, we employed the FRAP assay, which has been extensively utilized for assessing the total antioxidant content of various medicinal or food materials. The obtained FRAP values for different drying-treated lilies are presented in Figure 2A. The FRAP values of lily samples ranged from 3.63 to 5.01 mg Trolox^®^/g, exhibiting some variations depending on the employed drying treatments. Notably, VD-treated lilies exhibited higher FRAP values compared to those subjected to other drying treatments, indicating that a VD treatment contributes to an enhanced antioxidant capacity. A previous study reported higher FRAP values for vacuum-dried *Syzygium caryophyllatum* fruit compared to free-dried or hot-dried fruit [34]. This suggests that vacuum-drying treatment can enhance antioxidant capacity by facilitating the formation of new compounds with superior antioxidative properties than native compounds. Another study explained that the higher antioxidant activity in the VD samples may be ascribed to the increased generation of Maillard-type antioxidants [35]. Furthermore, a high correlation between the TPC and FRAP was observed, with the exception of the VD samples. Numerous studies have reported a strong correlation between the assays of TPC and antioxidant capacity, indicating that phenolic compounds are key contributors to the antioxidant capacity of herb extracts [36,37].

### 3.6. The Content of Polysaccharide and Alkaloid

The impact of different drying treatments on the polysaccharide content of lilies is depicted in Figure 2B. The polysaccharide content ranged from 40.24 to 73.56 mg/g. The results indicate significant variations in the polysaccharide content of lilies as a result of different drying treatments. Amongst the various drying treatments, VFD exhibited the highest concentration of polysaccharides in lilies, followed by VD, while HAD and ID yielded comparatively lower levels. This can be attributed to the intensified oxidation reaction during the HAD and ID processes, which accelerates sugar degradation, whereas VFD and CD benefit from a vacuum or low-temperature environment that mitigates sugar decomposition. Regarding the alkaloid level, it ranged from 4.70 to 12.48 mg/g across various drying treatments. Similar trends in alkaloid variations were observed as those for polysaccharides.

### 3.7. Metabolomics Analysis

To explore metabolites of lilies that may be influenced by different drying treatments, metabolomic analysis was performed in the present work. The metabolites of all dried lily samples were extracted and analyzed by means of LC-MS/MS both in positive ion mode and negative ion mode. In total, 232 metabolites were detected in the positive ion mode, while 284 metabolites were detected in the negative ion mode. The information regarding metabolites are listed in Table S1. To obtain a more comprehensive metabolic profile, the metabolite information of positive and negative ion modes was combined in this work. With respective to the metabolites detected by both modes, the mode with a large relative peak area was selected for analysis. Finally, 464 metabolites in positive/negative ion modes were obtained and used in the subsequent statistical analysis.

**Figure 3:**
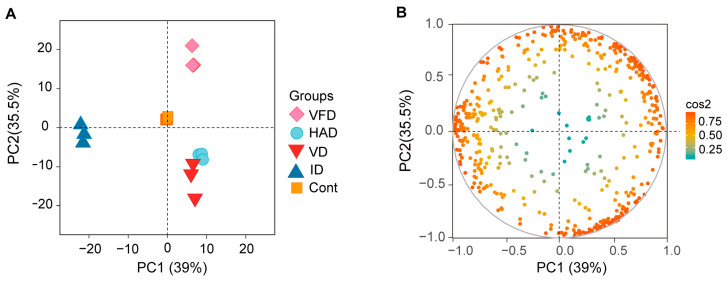
The PCA scores (**A**) and loading (**B**) plots of metabolite profiles in different drying-treated lily samples.

Multivariate statistical analysis was conducted to assess the differences among the metabolomic profiles of various dried lily samples. As a pre-analysis and quality control step for metabolomics data, unsupervised chemometrics analysis using principal component analysis (PCA) was employed to identify classification trends between groups and detect any potential outliers in the data. Figure 3 illustrates the distribution of samples on the PCA plot, which represents the first two principal components explaining 39% and 35.5% of the total variance, respectively. This figure demonstrates that QC samples, i.e., mixtures of different dried lily extracts, were clustered together, indicating instrument stability and reliable metabolomics data. Furthermore, the clustering of samples within each group revealed no significant differences between parallel samples. Samples obtained from different drying treatments formed separate clusters in the PCA scores plot, suggesting distinctive metabolic profiles for each group. Specifically, VFD-treated and ID-treated samples exhibited clear separation from other drying treatments in terms of their metabolic profiles. Conversely, HAD and VD showed high similarity with each other since their respective sample clusters were closely located on the PCA score plot. These results indicate that various drying treatments significantly influenced lilies’ metabolomic profiles.

**Figure 4:**
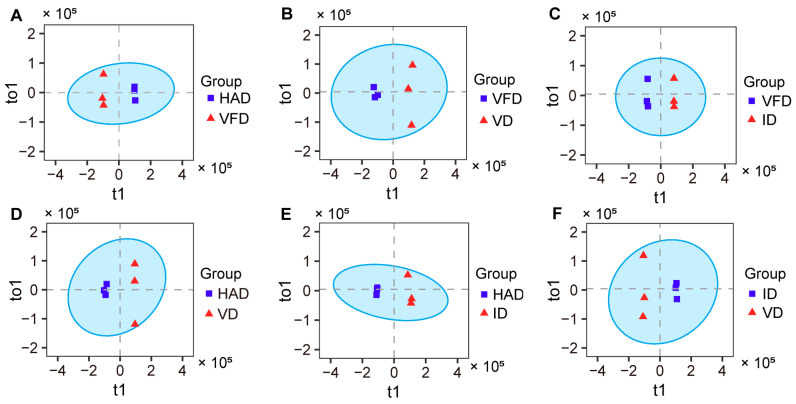
OPLS-DA score plot: (**A**) HAD vs. VFD; (**B**) VFD vs. VD; (**C**) VFD vs. ID; (**D**) HAD vs. VD; (**E**) HAD vs. ID; (**F**) ID vs. VD.

A supervised OPLS-DA approach was applied in the analysis of metabolite data. Pairwise comparisons were performed between different drying treatments, and the results are displayed in Figure 4. In the OPLS-DA models, two drying treatments were clearly separated. 

**Figure 5:**
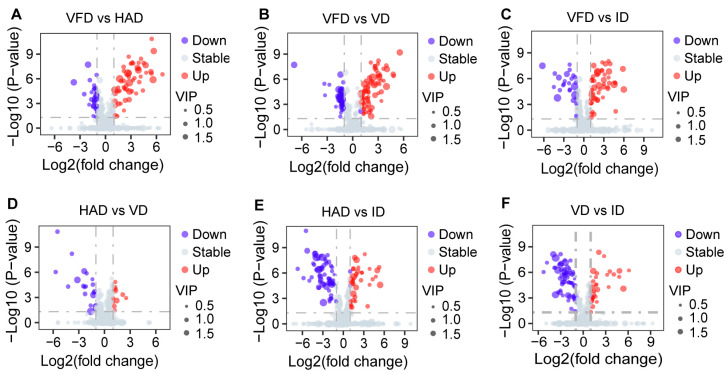
Volcano plot of the differential metabolites: (**A**) VFD vs. HAD; (**B**) VFD vs. VD; (**C**) VFD vs. ID; (**D**) HAD vs. VD; (**E**) HAD vs. ID; (**F**) VD vs. ID.

**Figure 6:**
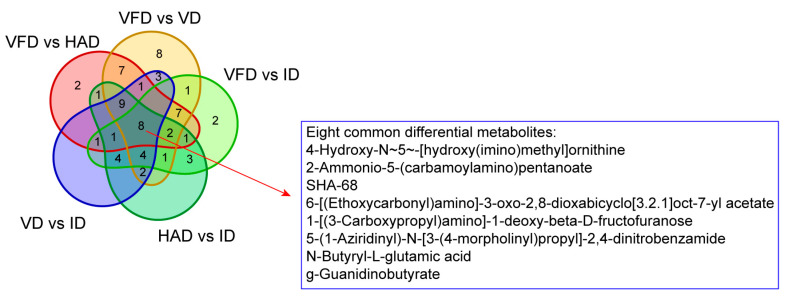
Venn plot indicating the number of common metabolites among the comparison groups.

The differential metabolites between the pairwise group were identified by the criteria of fold-change (FC ≥ 2 or ≤ 0.5), *p* value (*p* < 0.05), and variables with significant importance in the projection (VIP > 1) scores. The results of these screenings are presented as volcano plots in Figure 5. In comparison to VFD, HAD exhibited 40 differential metabolites (13 upregulated and 27 downregulated), VD showed 53 differential metabolites (22 upregulated and 31 downregulated), and ID had 35 differential metabolites (7 upregulated and 28 downregulated). When compared to ID, HAD displayed 36 differential metabolites (16 upregulated and 20 downregulated), while VD showed a total of 33 differential metabolites (13 upregulated and 20 downregulated). The smallest number of differential metabolites was observed in the HAD vs. VD group, with only 10 differential metabolites, suggesting a relatively smaller distinction between these groups in comparison with others. This finding is consistent with the results obtained from PCA; hence, subsequent differential analyses focused on other comparisons excluding the HAD vs. VD group. A Venn diagram representation illustrated commonalities among pairwise comparisons, as shown in Figure 6. A total of 150 differential metabolites existed across all pairwise comparisons. Among them, eight shared metabolites occurred involving amino acids and their derivatives.

**Figure 7:**
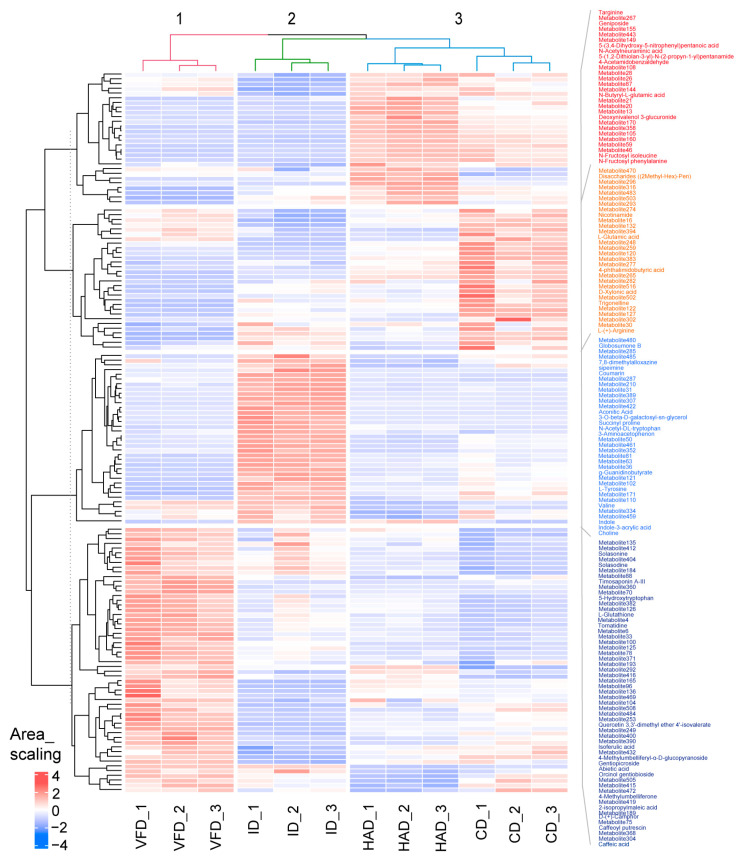
Hierarchical cluster analysis and heatmap of differential metabolite profiles of various drying-treated lilies.

The differences in the profiles of 150 differential metabolites across four drying treatments of lilies were further evaluated using heat map analysis combined with hierarchical clustering analysis (HCA). The results are displayed in Figure 7. Three clusters were obtained for the drying treatments, and HAD and VD formed a cluster. Thus, the HCA results coincided well with PCA. Additionally, it was indicated that VFD-dried lily samples exhibited the highest levels of steroids or saponin (solasonine, solasodine, timosaponin A-III, tomatidine, gentiopicroside, (3β,5α,17β)-6-oxocevan-3-yl β-D-glucopyranoside, etc.) and flavonoids or phenolic glycoside (quercetin 3,3′-dimethyl ether 4′-isovalerate, 4-methylumbelliferyl-α-D-glucopyranoside, orcinol gentiobioside, etc.). These compounds have been confirmed as key bioactive constituents in lilies, and thus the freeze-drying treatment is more effective in preserving these bioactive constituents. The ID treatment exhibited the highest content of several amino acids or derivatives, such as 4-aminobutanoate, succinyl proline, N-acetyl-dl-tryptophan, 1-[(3-carboxypropyl)amino]-1-deoxy-beta-D-fructofuranose, L-tyrosine, g-guanidinobutyrate, and valine. The HAD and VCD treatments had a relatively high content of other identified amino acids or derivatives, such as 2-ammonio-5-(carbamoylamino)pentanoate, N-butyryl-L-glutamic acid, 4-hydroxy-N~5~-[hydroxy(imino)methyl]ornithine, and dimethyl 4-amino-2-(2-methoxy-2-oxoethyl)-5-oxo-2,5-dihydro-1H-pyrrole-2,3-dicarboxylate. These results reveal that drying techniques exert a significant influence on the stability of amino acids and their derivatives.

The process of drying is widely employed as a technique for the preservation of lily bulbs. Several studies have stated that the quality attributes of products in terms of color, phytochemical components, and antioxidant ability are affected by drying technology employed. Huang et al. reported that prolonged exposure to high air temperatures or extended processing times can lead to quality changes in lily-bulb products, such as browning, oxidation, nutrition, and natural flavor [38]. Quan et al. found that some characteristic phytochemicals in lily bulbs, including Regaloside A, Regaloside B, and Regaloside E, were increased after special drying treatments [15]. Yuan et al. also demonstrated that different drying methods can affect the chemical constituents of the lily, and Regaloside A was higher in the VD group than in the HAD group [16]. In comparison with these previous studies, these characteristic phytochemicals were not identified in the present work. The reason could be that different lily varieties lead to certain differences in their chemical composition.

## 4. Conclusions

The present study investigated the effects of various drying treatments on the characteristics of lily bulbs. The results reveal significant variations in color, amino acid composition, TPC, TFC, and polysaccharide and alkaloid content among the various drying treatments. Importantly, VFD provided better preservation of original coloration and displayed the highest levels of total amino acid, TPC, TFC, and polysaccharide and alkaloid content. VD provided higher FRAP values compared to other drying treatments. Metabolomics analysis screened 150 differential metabolites, with the HCA indicating that VFD led to higher levels of steroids, saponin, flavonoids and phenolic glycoside. These findings suggested that VFD holds greater potential for preserving bioactive constituents in lily bulbs.

## Figures and Tables

**Table 1:** Humidity and drying time of dried lily samples.

Drying Method	Humidity (%)	Drying Time (h)
VFD	7.6 ± 0.3	15 h
HAD	8.2 ± 0.4	18 h
VD	8.7 ± 0.6	18 h
ID	8.9 ± 0.6	1 h

**Table 2:** Color parameters of dried lily samples.

Parameter	Fresh Lily	VFD	HAD	VD	ID
L*	82.35 ± 2.2	90.88 ± 1.0	67.83 ± 1.1 *	67.32 ± 0.2 *	69.98 ± 0.9 *
a*	0.24 ± 0.1	−1.52 ± 0.1	3.47 ± 0.2 *	3.05 ± 0.7 *	4.69 ± 0.6 *
b*	14.26 ± 0.5	12.77 ± 1.6	18.75 ± 2.0 *	19.78 ± 0.4 *	24.39 ± 0.7 *
∆*E*	/	8.84	14.22 *	16.26 *	16.60 *

* indicates significant difference when compared with VFD (*p* < 0.05).

**Table 3:** Free amino acids composition of dried lily sample.

Amino Acids	VFD	HAD	VD	ID
Aspartic acid (mg/g)	1.12 ± 0.05	1.16 ± 0.03	1.20 ± 0.03	0.90 ± 0.01 *
Glutamic acid (mg/g)	3.35 ± 0.06	3.48 ± 0.05 *	3.45 ± 0.05	2.87 ± 0.03 *
Serine (mg/g)	0.43 ± 0.01	0.39 ± 0.01 *	0.34 ± 0.01 *	0.24 ± 0.01 *
Glycine (mg/g)	0.54 ± 0.01	0.42 ± 0.01 *	0.49 ± 0.01 *	0.39 ± 0.01 *
Histidine (mg/g)	0.25 ± 0.01	0.16 ± 0.01 *	0.13 ± 0.01 *	0.12 ± 0.01 *
Arginine (mg/g)	7.93 ± 0.09	5.20 ± 0.06 *	5.59 ± 0.06 *	6.89 ± 0.05 *
Threonine (mg/g)	0.04 ± 0.00	0.03 ± 0.00	0.03 ± 0.00	0.05 ± 0.00
Alanine (mg/g)	0.14 ± 0.01	0.15 ± 0.01	0.12 ± 0.01	0.13 ± 0.01
Proline (mg/g)	0.09 ± 0.00	0.06 ± 0.00	0.10 ± 0.00	0.12 ± 0.00
Tyrosine (mg/g)	0.04 ± 0.00	0.03 ± 0.00	0.03 ± 0.00	0.07 ± 0.00
Valine (mg/g)	0.20 ± 0.00	0.15 ± 0.00	0.15 ± 0.00	0.25 ± 0.00
Methionine (mg/g)	0.09 ± 0.00	0.08 ± 0.00	0.08 ± 0.00	0.04 ± 0.00
Cystine (mg/g)	ND	ND	ND	ND
Isoleucine (mg/g)	0.02 ± 0.00	0.02 ± 0.00	0.02 ± 0.00	0.01 ± 0.00
Leucine (mg/g)	1.75 ± 0.01	0.91 ± 0.01 *	1.10 ± 0.01 *	1.67 ± 0.01 *
Phenylalanine (mg/g)	0.18 ± 0.00	0.08 ± 0.00	0.10 ± 0.00	0.14 ± 0.00
Lysine (mg/g)	0.44 ± 0.01	0.41 ± 0.02	0.39 ± 0.01 *	0.25 ± 0.01 *
Total amino acids (mg/g)	16.60	12.75	13.33	14.14

* indicates significant difference when compared with VFD (*p* < 0.05). ND denotes not detected.

## Data Availability

The original contributions presented in the study are included in the article/Supplementary Material, further inquiries can be directed to the corresponding authors.

## Supplementary Materials

The following supporting information can be downloaded at: https://www.mdpi.com/article/10.3390/foods13142206/s1, Table S1. The information of metabolites.
