# Supplementary material for: A Comparison Analysis of Quality and Metabolic Compounds in Lilies with Different Drying Treatments
Source: Foods. 2024 Jul 13;13(14):2206. doi: 10.3390/foods13142206 (PMC11275255; doi:10.3390/foods13142206)
Supplement: Supplementary file 1 [file foods-13-02206-s001.zip › Supplementary_Material.pdf]

Supplementary Material

A comparison analysis of quality and metabolic compounds in lilies with different drying treatments

Lixia Xie[1,2], Jiajia Liu[1,2], Haoyu Wu[1,2], Yueyan Zhong[1,2], Xueying Liu[1,2], Guangli Li[2*], Xizhu Lin[3], Laijun Xu[3] and Zhi Liu[4*]

*** Correspondence:** Guangli Li: guangli010@163.com; Zhi Liu: liuzhi_schoolar@126.com

1 Supplementary Data

Table S1 The information of metabolites

Metabolite Number	Metabolite Name
Metabolite1	L-(+)-Arginine
Metabolite2	4-Coumaric acid
Metabolite3	Leucine
Metabolite4	3,12,21,24,25-Pentahydroxylanost-8-en-2-yl 3-hydroxy-5-[(2-methoxy-2-oxoethyl)amino]-3-methyl-5-oxopentanoate
Metabolite5	Tomatidine
Metabolite6	(3beta,16alpha,17beta)-16-Hydroxy-17-{(1S)-1-[(2R,5S)-5-methyl-2-piperidinyl]ethyl}androst-5-en-3-yl beta-D-glucopyranoside
Metabolite7	Methyl N-(4-{[7-(4-{[(2S,3R)-3-hydroxy-1-{[(2S)-1-methoxy-4-methyl-1-oxo-2-pentanyl]amino}-1-oxo-2-butanyl]amino}-4-oxobutoxy)-2-naphthyl]oxy}butanoyl)-L-valyl-L-leucyl-L-valinate
Metabolite8	N-Hydroxysuccinimide
Metabolite9	L-Glutamic acid
Metabolite10	p-Coumaraldehyde
Metabolite11	L-Glutamine
Metabolite12	(3beta,5beta,22R)-14-Hydroxy-6-oxocholest-7-ene-3,22,25-triyl triacetate
Metabolite13	N-{1-[(4-Morpholinylcarbonyl)amino]ethyl}-alpha-asparagine
Metabolite14	L-Pyroglutamic acid
Metabolite15	(3beta,5alpha,25R)-12-Oxospirost-9(11)-en-3-yl 6-deoxy-alpha-L-mannopyranosyl-(1->2)-[6-deoxy-alpha-L-mannopyranosyl-(1->4)]-beta-D-glucopyranoside
Metabolite16	2-Furoic acid
Metabolite17	N,N'-Bis(2-methoxyethyl)-4,6-dinitro-1,3-benzenediamine
Metabolite18	N-({5-[6-(2-Amino-4-oxo-4,7-dihydro-3H-pyrrolo[2,3-d]pyrimidin-5-yl)hexyl]-2-thienyl}carbonyl)-L-glutamic acid
Metabolite19	Indole-3-acrylic acid
Metabolite20	4-Hydroxy-N~5~-[hydroxy(imino)methyl]ornithine
Metabolite21	2-Ammonio-5-(carbamoylamino)pentanoate
Metabolite22	2-Hydroxycinnamic acid

Metabolite23	(1S,3R,5R,6aS,7R,8R,10S,10aS)-1,3-Diacetoxy-7-[(2S)-2-hydroxy-3-methylene-4-penten-1-yl]-7,8-dimethyl-10-(D-xylopyranosyloxy)-3,5,6,6a,7,8,9,10-octahydronaphtho[1,8a-c]furan-5-yl decanoate
Metabolite24	Trigonelline
Metabolite25	5'-S-Methyl-5'-thioadenosine
Metabolite26	(5R,6S,7aS)-6-Hydroxy-5-(hydroxymethyl)tetrahydro-1H-pyrrolo[1,2-c][1,3]oxazol-3-one
Metabolite27	N-Cyclopentyl-2-(phenylsulfonyl)-9-(D-ribofuranosyl)-9H-purin-6-amine
Metabolite28	3-(2-Oxo-2,5-dihydro-3-furanyl)propanoic acid
Metabolite29	2-amino-2,3,7-trideoxy-D-lyxo-hept-6-ulosonic acid
Metabolite30	2-Isopropyl-5-thieno[3,2-b]thiophen-2-yl-1,3,4-oxadiazole
Metabolite31	4-AMINOBUTANOATE
Metabolite32	4-(1,2-Dihydroxyethyl)-2,6-piperidinedione
Metabolite33	3-O-alpha-L-Allopyranosyl-2,6-anhydro-5-deoxy-D-xylo-hex-5-enitol
Metabolite34	Valine
Metabolite35	(3R)-3-Hydroxy-5-{[2-({N-[(2R)-2-hydroxy-3-methylbutanoyl]-beta-alanyl}amino)ethyl]amino}-5-oxopentanoic acid
Metabolite36	2,5-Anhydro-6-O-(5-carbamimidamido-5-deoxy-beta-D-ribofuranosyl)-1-deoxy-1-(2,4-dioxo-3,4-dihydro-1(2H)-pyrimidinyl)-D-glucitol
Metabolite37	L-Histidine
Metabolite38	(2S,3R,4R,5R,6S)-2-{[(2R,3R,4S,5S,6R)-4,5-dihydroxy-6-(hydroxymethyl)-2-{5',7,9,13-tetramethyl-5-oxaspiro[pentacyclo-icosane-6,2'-piperidine]-3-oloxy}oxan-3-yl]oxy}-6-methyloxane-3,4,5-triol
Metabolite39	L-Glutathione (reduced)
Metabolite40	L-Aspartic acid
Metabolite41	Ethylene glycol diacetate
Metabolite42	Hecogenin
Metabolite43	N-(Phenylcarbamoyl)-5'-O-sulfamoyladenosine
Metabolite44	SOLASODINE
Metabolite45	Hupehemonoside
Metabolite46	(2R,6S)-2-Amino-6-(D-gamma-glutamylamino)heptanedioic acid
Metabolite47	Choline
Metabolite48	3-Aminoacetophenon
Metabolite49	DL-Metanephrine
Metabolite50	3,5,7-Octatriyn-1-ol
Metabolite51	Butyl (3beta,5alpha,7beta,12beta)-12-acetoxy-3,7-dihydroxy-4,4,14-trimethyl-11,15-dioxochol-8-en-24-oate
Metabolite52	3,3'-(3,8,13,17-Tetramethyl-7,12-divinyl-2,3,6,7,8,11,12,13,14,17,22,24-dodecahydroporphyrin-2,18-diyl)dipropanoic acid
Metabolite53	7-Hydroxy-5,4'-dimethoxyflavone 8-C-rhamnoside
Metabolite54	3-Methylsulfolene
Metabolite55	Caffeic acid
Metabolite56	Gracillin
Metabolite57	7-Hydroxycoumarine
Metabolite58	(1S,2S,3R)-N-(2,4-Dichlorobenzyl)-2-methyl-3-phenylcyclopropanamine
Metabolite59	Methyl {(2Z)-3-[4,6-bis(dimethylamino)-1,3,5-triazin-2-yl]-2-imino-5-oxo-1-imidazolidinyl}acetate
Metabolite60	Targinine

Metabolite61	Isoferulic acid
Metabolite62	Proline
Metabolite63	SHA-68
Metabolite64	N-[1-Cyclohexyl-5-oxo-2-phenyl-4-(trifluoromethyl)-4,5-dihydro-1H-imidazol-4-yl]-2-fluorobenzamide
Metabolite65	Gentiopicroside
Metabolite66	Quercetin 3,3'-dimethyl ether 4'-isovalerate
Metabolite67	alpha-Glutamyl-4-hydroxyproline
Metabolite68	6-Oxo-pipecolinic acid
Metabolite69	4-Methylumbelliferyl-α-D-glucopyranoside
Metabolite70	Primobolan
Metabolite71	D-(+)-Maltose
Metabolite72	Pipecolic acid
Metabolite73	3-({4-[(4-Amino-1H-pyrazolo[3,4-d]pyrimidin-1-yl)methyl]-1H-1,2,3-triazol-1-yl}methoxy)-1,2-propanediol
Metabolite74	L-Homopropargyl Glycine
Metabolite75	(3beta,5alpha,17beta)-6-Oxocevan-3-yl beta-D-glucopyranoside
Metabolite76	Solasonine
Metabolite77	Muramic acid
Metabolite78	O-(2-Methyl-2-propanyl)-N-{[(2-methyl-2-propanyl)oxy]carbonyl}-L-seryl-D-leucyl-L-phenylalanyl-D-leucyl-L-phenylalanine
Metabolite79	Khasianine
Metabolite80	Dicoumaroyl Spermidine
Metabolite81	2-Acetyl-1H-pyrrol-3-yl 6-deoxy-2-O-(6-deoxy-beta-D-glucopyranosyl)-beta-D-glucopyranoside
Metabolite82	Feruloyl putrescine (isomer of 1178)
Metabolite83	Bis(4-ethylbenzylidene)sorbitol
Metabolite84	N-Acetyl-L-seryl-L-alpha-aspartyl-L-ornithyl-L-proline
Metabolite85	(2S)-2-[(2-Amino-2-methylpropyl)amino]-3-(4-biphenylyl)-N-[(1S)-1-(5,6-dichloro-1H-benzimidazol-2-yl)ethyl]propanamide
Metabolite86	Phosphinic acid, P-(1-aminopentyl)-P-[[[(4-methoxyphenyl)methyl]amino]thioxomethyl]-
Metabolite87	6-[(Ethoxycarbonyl)amino]-3-oxo-2,8-dioxabicyclo[3.2.1]oct-7-yl acetate
Metabolite88	Methyl (4S)-4-[(5''R,10''S,12''S,13''R,17''R)-12''-acetoxy-10'',13''-dimethylhexadecahydrodispiro[cyclohexane-1,3'-[1,2,4,5]tetroxane-6',3''-cyclopenta[a]phenanthren]-17''-yl]pentanoate
Metabolite89	N~2~-(1-Carboxyethyl)-N~5~-(diaminomethylene)ornithine
Metabolite90	Nicotinamide
Metabolite91	6-Acetamido-2-oxohexanoic acid
Metabolite92	L-Tyrosine
Metabolite93	dimethyl 3,3'-heptadec-1-ene-1,1-diylbis(5-bromo-6-methoxybenzoate)
Metabolite94	Arbutin
Metabolite95	Argininosuccinic acid
Metabolite96	(2S)-1-{[(2S,3S,5R)-6-(Butylamino)-1-cyclohexyl-3-hydroxy-5-methyl-6-oxo-2-hexanyl]amino}-1-oxo-2-hexanyl hydrogen (3-phenylpropyl)phosphonate
Metabolite97	4-Aminophenol
Metabolite98	Testosterone cypionate

Metabolite99	(3S,6S,9R,10R,11S,12S,15E,18S,21S)-10,12-Dihydroxy-3-(3-hydroxybenzyl)-6-isopropyl-11-methyl-9-(3-oxobutyl)-18-[(2E)-2,4-pentadien-2-yl]-19-oxa-1,4,7,25-tetraazabicyclo[19.3.1]pentacosa-13,15-diene-2,5,8,20-tetrone
Metabolite100	NCGC00179940-02_C39H64O13_beta-D-Glucopyranoside, 3,23-dihydroxyspirostan-6-yl 6-deoxy-4-O-(6-deoxy-alpha-L-mannopyranosyl)-
Metabolite101	(5alpha,21R,24R)-17,21-Dihydroxy-21,24-epoxycholestan-3-one
Metabolite102	1-[(3-Carboxypropyl)amino]-1-deoxy-beta-D-fructofuranose
Metabolite103	Coumaroyl putrescin
Metabolite104	1-{5-(Methylsulfonyl)-3-[3-{[2-(1-piperidinyl)ethyl]sulfanyl}-4-(trifluoromethyl)phenyl]-4,5,6,7-tetrahydro-1H-pyrazolo[4,3-c]pyridin-1-yl}-3-(1-pyrrolidinyl)-2-propanol
Metabolite105	(6R)-5-Acetamido-2,6-anhydro-3,5-dideoxy-6-[(1S)-1,2,3-trihydroxypropyl]-L-threo-hex-2-enonic acid
Metabolite106	Timosaponin A-III
Metabolite107	(3beta,5alpha,25R)-12-Oxospirostan-3-yl 2-O-(6-deoxy-alpha-L-mannopyranosyl)-beta-D-glucopyranoside
Metabolite108	5-(1-Aziridinyl)-N-[3-(4-morpholinyl)propyl]-2,4-dinitrobenzamide
Metabolite109	L-(-)-Threonine
Metabolite110	N~5~-(4-Methyl-5-oxo-4,5-dihydro-1H-imidazol-2-yl)-L-ornithine
Metabolite111	8-(3-Chloro-4-fluorobenzyl)-2,3,4,6-tetrahydropyrido[2,3-d]pyridazin-5(1H)-one
Metabolite112	Methyl (9aS,10S)-10,13-dihydroxy-15-methoxy-4,14-dioxo-10,11,12,14-tetrahydro-4H-chromeno[2,3-j][1,2]oxazolo[4,3,2-de]phenanthridine-9a(7H)-carboxylate
Metabolite113	N-Acetylneuraminic acid
Metabolite114	(9alpha,13alpha,16alpha,17alpha,20S)-25-Butoxy-1,16,20,22-tetrahydroxy-9,14-dimethyl-11-oxo-4,9-cyclo-9,10-secocholesta-1,3,5(10),23-tetraen-2-yl hexopyranoside
Metabolite115	N-Butyryl-L-glutamic acid
Metabolite116	(1R,2S,3R)-1-[2-(3-Methyl-1,2-oxazol-5-yl)-1H-imidazol-4-yl]-1,2,3,4-butanetetrol
Metabolite117	9-[4-(5-{[2-(1,3-Benzoxazol-2-yl)benzoyl]amino}-2-oxido-1,3,2-dioxaphosphinan-2-yl)butyl]-N-(2,2,2-trifluoroethyl)-9H-fluorene-9-carboxamide
Metabolite118	4-Hydroxybenzaldehyde
Metabolite119	2,5-Dimethylfuran-3(2H)-one
Metabolite120	Laurixamine
Metabolite121	N-(2,5-Dimethoxyphenyl)-2,3-diphenyl-6-quinoxalinecarboxamide
Metabolite122	pilaralisib
Metabolite123	Protirelin
Metabolite124	2-(1,3-Dihydroxy-4-oxocyclohexyl)-5-hydroxy-3,6,7-trimethoxy-4H-chromen-4-one
Metabolite125	3-O-ETHYL ASCORBIC ACID
Metabolite126	NCGC00384584-01_C51H82O23_(3beta,9xi,22S,25S)-26-(beta-D-Glucopyranosyloxy)-22,25-epoxyfurost-5-en-3-yl 6-deoxy-alpha-L-mannopyranosyl-(1->4)-beta-D-glucopyranosyl-(1->2)-beta-D-glucopyranoside
Metabolite127	(2S)-2-{[{[(2R,3S,5R)-5-(4-Amino-2-oxo-1(2H)-pyrimidinyl)-3-hydroxytetrahydro-2-furanyl]methoxy}(hydroxy)phosphoryl]amino}succinic acid

Metabolite128	N-(4-Benzoylphenyl)-3-methylbenzamide
Metabolite129	(4aR,5R,10bR)-1-{[{8-[(4-fluorobenzyl)(methyl)amino]octyl}(methyl)amino]acetyl}-12-methyl-2,3,4,4a,5,6-hexahydro-1H-5,10b-prop[1]eno-1,7-phenanthrolin-8(7H)-one
Metabolite130	Coumarin
Metabolite131	2,6-Dimethyl-γ-pyrone
Metabolite132	4-(1-{[(2S,3S,4R)-3,4-Dihydroxy-2-pyrrolidinyl]methyl}-1H-1,2,3-triazol-4-yl)butanoic acid
Metabolite133	4-Guanidinobutyric acid
Metabolite134	2-(Benzyloxy)ethyl {(1R,2R,6S,6aS,11bS,11cS)-6a-(allyloxy)-4-(ethoxyimino)-1,2-bis(4-hydroxybutyl)-10-[(6-methyl-2-pyridinyl)methoxy]-1,2,4,5,6,6a,11b,11c-octahydrobenzo[kl]xanthen-6-yl}[2-(2-hydroxyethoxy)ethyl]carbamate
Metabolite135	(2S)-N-tert-butyl-1-[(2S,4R)-4-(cyclohexylmethyl)-2-hydroxy-5-{[(1S,2R)-2-hydroxy-2,3-dihydro-1H-inden-1-yl]amino}-5-oxopentyl]-4-(pyridin-3-ylmethyl)piperazine-2-carboxamide (non-preferred name)
Metabolite136	(3beta,5beta,25S)-17-Hydroxyspirostan-3-yl 2-O-alpha-L-altropyranosyl-beta-D-glucopyranoside
Metabolite137	g-Guanidinobutyrate
Metabolite138	N-Fructosyl isoleucine
Metabolite139	Methyl 2-acetamido-2-deoxyhexopyranoside
Metabolite140	Diethyl acetamidomalonate
Metabolite141	Eglumetad
Metabolite142	4-Indolecarbaldehyde
Metabolite143	(2Z)-2-[4-({[2-(4-Methyl-1-piperazinyl)ethyl]sulfonyl}amino)benzylidene]-3-oxo-2,3-dihydro-1-benzofuran-7-carboxamide
Metabolite144	N-(1-Hydroxy-2-butanyl)-2-(methylsulfanyl)-N-(2-thienylmethyl)acetamide
Metabolite145	(2S)-2-[(3-Carboxypropanoyl)amino]hexanedioic acid
Metabolite146	4-[(4-Nitro-2,1,3-benzoxadiazol-5-yl)amino]benzamide
Metabolite147	PMK ethyl glycidate
Metabolite148	Indole
Metabolite149	N-(4-Acetamidophenyl)-1-methyl-4-nitro-1H-pyrazole-5-carboxamide
Metabolite150	NCGC00180097-02!
Metabolite151	4-Amino-N'-[(E)-(3,4,5-trimethoxyphenyl)methylene]-1,2,5-oxadiazole-3-carbohydrazide
Metabolite152	4-Formylphenyl beta-D-ribopyranoside
Metabolite153	2-(Hydroxymethyl)-6-{(1R)-1-hydroxy-2-[(2-methyl-2-propanyl)amino]ethyl}-3-pyridinol
Metabolite154	(3S,6S,9R,14R,19aR)-14-Amino-3-benzyl-6-isopropyl-1,4,7,15-tetraoxohexadecahydro-13H-pyrrolo[2,1-m][1,2,5,8,11,14]dithiatetraazacycloheptadecine-9-carboxylic acid
Metabolite155	Pyridoxine + O-Hex
Metabolite156	(1beta,3beta,5alpha,25R)-3-Hydroxyspirostan-1-yl beta-D-glucopyranoside
Metabolite157	(1R,2S,5S)-N-[4-(Allylamino)-1-cyclobutyl-3,4-dioxo-2-butanyl]-3-(N-{[(2R)-1-(4,4-dimethyl-2,6-dioxo-1-piperidinyl)-3,3-dimethyl-2-butanyl]carbamoyl}-3-methyl-L-valyl)-6,6-dimethyl-3-azabicyclo[3.1.0]hexane-2-carboxamide
Metabolite158	1-[(E)-Amino{[3-(3,4,5-trimethoxyphenoxy)propoxy]amino}methylene]-2-isopropylguanidine
Metabolite159	L-2-succinylamino-6-oxoheptanedioic acid

Metabolite160	Dimethyl 4-amino-2-(2-methoxy-2-oxoethyl)-5-oxo-2,5-dihydro-1H-pyrrole-2,3-dicarboxylate
Metabolite161	(2E)-4-(Cyclopropylamino)-4-oxo-2-butenoic acid
Metabolite162	Succinyl proline
Metabolite163	4-phthalimidobutyric acid
Metabolite164	4-Acetylphenyl 6-O-beta-D-xylopyranosyl-beta-D-glucopyranoside
Metabolite165	delta4-Dafachronic acid
Metabolite166	(2S)-Amino(2-furyl)acetic acid
Metabolite167	Ethyl alpha-D-glucopyranoside
Metabolite168	Adenine
Metabolite169	Nicotinic acid
Metabolite170	trans-Ferulic acid; [M+H-H2O]+
Metabolite171	LPC 18:2
Metabolite172	(3aS,4aR,7aS,9bS)-3-Ethyl-2-oxo-3,3a,7a,9b-tetrahydro-2H,4aH-1,4,5-trioxadicyclopenta[a,hi]indene-7-carboxylic acid
Metabolite173	sipeimine
Metabolite174	6-Methylquinoline
Metabolite175	16-{[(2R,3R,4S,5S,6R)-4,5-dihydroxy-6-(hydroxymethyl)-3-{[(2S,3R,4R,5R,6S)-3,4,5-trihydroxy-6-methyloxan-2-yl]oxy}oxan-2-yl]oxy}-5',7,9,13-tetramethyl-5-oxaspiro[pentacyclo[10.8.0.0?,?.0?,?.0??,??]icosane-6,2'-piperidine]-3-one
Metabolite176	Petiline
Metabolite177	Prolylleucine
Metabolite178	D-(+)-Camphor
Metabolite179	4-Hydroxycoumarin
Metabolite180	isoverticine
Metabolite181	Adenosine
Metabolite182	3-Succinoylpyridine
Metabolite183	Methyl (3S,3aS,4aR,7aS,9aS,9bS)-3-ethyl-2-oxo-3,3a,7a,9b-tetrahydro-2H,4aH-1,4,5-trioxadicyclopenta[a,hi]indene-7-carboxylate
Metabolite184	NCGC00385395-01_C51H84O23_(3beta,22R,25R)-26-(beta-D-Glucopyranosyloxy)-22-hydroxyfurost-5-en-3-yl 6-deoxy-alpha-L-mannopyranosyl-(1->2)-[beta-D-glucopyranosyl-(1->4)]-beta-D-glucopyranoside
Metabolite185	Mebeverine
Metabolite186	N-Fructosyl phenylalanine
Metabolite187	Pennogenin 3-O-β-chacotrioside
Metabolite188	Caffeoyl putrescin (isomer of 1060)
Metabolite189	D-(-)-Salicin
Metabolite190	4-Methylumbelliferone
Metabolite191	Phthaldialdehyde
Metabolite192	LPE 18:2
Metabolite193	3-hydroxy-3-methyl-5-oxo-5-[[(2R,3S,4S,5R,6S)-3,4,5-trihydroxy-6-(2-methyl-4-oxopyran-3-yl)oxyoxan-2-yl]methoxy]pentanoic acid
Metabolite194	Quercetin-3β-D-glucoside
Metabolite195	5-Hydroxytryptophan
Metabolite196	(3aR,4aS,5R,7aS,8S,9aR)-5-Hydroxy-4a,8-dimethyl-3-methyleneoctahydroazuleno[6,5-b]furan-2,6(3H,4H)-dione

Metabolite197	Sinapoyl + C6H9O5
Metabolite198	3-n-Butylphathlide
Metabolite199	Sinapinic acid
Metabolite200	2-[(3S)-1-(Cyclohexylmethyl)-3-pyrrolidinyl]-5-(1-methyl-1H-imidazol-5-yl)-1,3,4-oxadiazole
Metabolite201	7-Methylsulfenylheptyl isothiocyanate
Metabolite202	6-Methylindole
Metabolite203	Ruscogenin
Metabolite204	NCGC00385600-01_C13H22O3_2-Cyclohexen-1-one, 4-hydroxy-4-(3-hydroxybutyl)-3,5,5-trimethyl-
Metabolite205	(7R,8S)-7,8-Dihydroxy-3,7-dimethyl-6-oxo-7,8-dihydro-6H-isochromene-5-carbaldehyde
Metabolite206	1-(4-Methylphenyl)pyrrolidine-2,5-dione
Metabolite207	Sarsasapogenin
Metabolite208	Quinoline
Metabolite209	delta-Decalactone
Metabolite210	Diethyl phthalate
Metabolite211	PC(16:0/0:0)
Metabolite212	Dehydrodiisoeugenol
Metabolite213	4-Acetamidobenzaldehyde
Metabolite214	Cinnamic acid
Metabolite215	Xanthorhamnin
Metabolite216	Kaempferol-7-O-glucoside
Metabolite217	Abietic acid
Metabolite218	Progesterone
Metabolite219	2-Hydroxyphenylalanine
Metabolite220	Apocynin
Metabolite221	Glutamylphenylalanine (isomer of 1503)
Metabolite222	5-Hydroxy-7-(hydroxymethyl)-2-methyl-2-(5-oxotetrahydro-2-furanyl)-2,3-dihydro-4H-chromen-4-one
Metabolite223	"4,7-dihydroxymellein_130075"
Metabolite224	Quercetin
Metabolite225	Rutin
Metabolite226	Stearamide
Metabolite227	1-palmitoyl-2-hydroxy-sn-glycero-3-phosphoethanolamine
Metabolite228	7,8-dimethylalloxazine (lumichrome)
Metabolite229	Jacein
Metabolite230	trans,trans-1,3-Bis(4-methoxybenzylidene) acetone
Metabolite231	3-Heptanone, 7-(3,4-dihydroxyphenyl)-5-hydroxy-1-(4-hydroxyphenyl)-
Metabolite232	(5alpha,6alpha)-17-(Cyclobutylmethyl)-4,5-epoxymorphinan-3,6,14-triol
Metabolite233	α,α-Trehalose
Metabolite234	Citric acid
Metabolite235	2-Oxoglutaric acid
Metabolite236	3-Buten-1-yl {(1R,2R,6S,6aS,11bS,11cS)-6a-(allyloxy)-1,2-bis(4-hydroxybutyl)-10-[(6-methyl-2-pyridinyl)methoxy]-4-[(tetrahydro-2H-pyran-2-yloxy)imino]-1,2,4,5,6,6a,11b,11c-octahydrobenzo[kl]xanthen-6-yl}(4-fluorobenzyl)carbamate
Metabolite237	1-[(4-Aminophenyl)sulfonyl]-L-prolyl-3-[(benzylcarbamoyl)amino]-L-alanine

Metabolite238	(17beta)-3-Hydroxy-2-iodoestra-1(10),2,4-trien-17-yl acetate
Metabolite239	2-Ketoadipic acid
Metabolite240	Methyl N-(4-{[7-(4-{[(2S,3R)-3-hydroxy-1-{[(2S)-1-methoxy-4-methyl-1-oxo-2-pentanyl]amino}-1-oxo-2-butanyl]amino}-4-oxobutoxy)-2-naphthyl]oxy}butanoyl)-L-valyl-L-leucyl-L-valinate
Metabolite241	Bis(hydroxymethyl)malonic acid
Metabolite242	N-[1-(4-Fluorobenzyl)-3,5-dimethyl-1H-pyrazol-4-yl]-5-(2-thienyl)-7-(trifluoromethyl)-4,5,6,7-tetrahydropyrazolo[1,5-a]pyrimidine-3-carboxamide
Metabolite243	4-(2-Chlorophenyl)-7-{[3-(4-hydroxytetrahydro-2H-pyran-4-yl)benzyl]oxy}naphtho[2,3-c]furan-1(3H)-one
Metabolite244	1-Deoxy-1-{2,6,8-trioxo-7-[3-(phosphonooxy)propyl]-1,2,3,6,7,8-hexahydro-9H-purin-9-yl}-D-ribitol
Metabolite245	(2E)-3-(4-Chlorophenyl)-N-[17-(cyclopropylmethyl)-6-oxo-4,5-epoxymorphinan-14-yl]acrylamide
Metabolite246	DL-Arginine
Metabolite247	(2S)-({[5-(2,6-Dimethoxyphenyl)-1-(4-fluorophenyl)-1H-pyrazol-3-yl]carbonyl}amino)(phenyl)acetic acid
Metabolite248	4-Ethoxy-N-{4-[4-(methylsulfonyl)-1-piperazinyl]phenyl}-3-nitrobenzamide
Metabolite249	Diferuloyl glycerol
Metabolite250	2-[(6-Amino-1-benzyl-2,4-dioxo-1,2,3,4-tetrahydro-5-pyrimidinyl)(isobutyl)amino]-2-oxoethyl (4-methyl-2-oxo-1,3-thiazol-3(2H)-yl)acetate
Metabolite251	Pennogenin 3-O-β-chacotrioside
Metabolite252	Ascorbic acid
Metabolite253	6,4'-Dihydroxy-7-methylaurone 6-rhamnoside
Metabolite254	(2,5-Dioxodihydroimidazo[4,5-d]imidazole-1,3,4,6(2H,5H)-tetrayl)tetrakis(methylene) tetraacetate
Metabolite255	Methyl N-[(4-chloro-3-quinolinyl)carbonyl]-S-methyl-L-cysteinate
Metabolite256	2-Isopropylmalic acid
Metabolite257	Coumaroyl Hexoside (isomer of 691, 692)
Metabolite258	(2R,3R,4S,5R,6R)-4-Hydroxy-5-{[(2S,3R,4S,5R,6R)-5-hydroxy-6-(hydroxymethyl)-4-{[(2S,3S,4R,5R,6R)-3,4,5,6-tetrahydroxytetrahydro-2H-pyran-2-yl]oxy}-3-{[(2S,3R,4S,5S,6R)-3,4,5-trihydroxy-6-(hydroxymethyl)tetrahydro-2H-pyran-2-yl]oxy}tetrahydro-2H-pyran-2-yl]oxy}-6-(hydroxymethyl)-2-{[(2S,4aR,4bS,5'S,6aS,6bR,7S,8R,9aS,10aS,10bS)-4a,5',6a,7-tetramethyl-1,2,3,3',4,4',4a,4b,5,5',6,6',6a,6b,7,9a,10,10a,10b,11-icosahydrospiro[naphtho[2',1':4,5]inde
Metabolite259	7-(Diethylamino)-2-oxo-N'-(2-thienylcarbonyl)-2H-chromene-3-carbohydrazonamide
Metabolite260	Sesaminol triglucoside
Metabolite261	D-(-)-Salicin
Metabolite262	(2S,3S,4S)-2-{(1R)-2-Amino-1-[(2S,3S,4R,5R)-5-(2,4-dioxo-3,4-dihydro-1(2H)-pyrimidinyl)-4-hydroxy-3-methoxytetrahydro-2-furanyl]-2-oxoethoxy}-3,4-dihydroxy-N-(2-hydroxyethyl)-3,4-dihydro-2H-pyran-6-carboxamide
Metabolite263	D-Xylulosonic acid
Metabolite264	2-[4-(Difluoromethoxy)-3-methoxyphenyl]-2-oxoethyl 1-[5-(trifluoromethyl)-2-pyridinyl]-4-piperidinecarboxylate
Metabolite265	Cabotegravir
Metabolite266	N-(Phenylcarbamoyl)-5'-O-sulfamoyladenosine

Metabolite267	4-Acetylphenyl 6-O-beta-D-xylopyranosyl-beta-D-glucopyranoside
Metabolite268	L-Phenylalanine
Metabolite269	1-O-[(2alpha,3beta)-2,20,23-Trihydroxy-28-oxo-3-{[3-O-(beta-D-xylopyranosyl)-alpha-L-arabinopyranosyl]oxy}urs-12-en-28-yl]-beta-D-glucopyranose
Metabolite270	NCGC00380914-01_C32H38O17_alpha-D-Glucopyranoside, 3,6-bis-O-[(2E)-3-(4-hydroxy-3-methoxyphenyl)-1-oxo-2-propen-1-yl]-beta-D-fructofuranosyl
Metabolite271	Hexose
Metabolite272	1-{(1S,2S)-4,6-Dichloro-1-[4-(3,5-dimethyl-4H-1,2,4-triazol-4-yl)phenoxy]-2,3-dihydro-1H-inden-2-yl}-1,4-diazepane
Metabolite273	1-Benzyl-2'-(2-furyl)-1',10b'-dihydrospiro[indole-3,5'-pyrazolo[1,5-c][1,3]benzoxazin]-2(1H)-one
Metabolite274	(3alpha,4beta,12xi)-3-(Tetrahydro-2H-pyran-2-yloxy)-12,13-epoxytrichothec-9-ene-4,15-diyl bis(chloroacetate)
Metabolite275	N-({5-[(2-{[2-Chloro-5-(trifluoromethyl)phenyl]amino}-2-oxoethyl)sulfanyl]-4-(4-nitrophenyl)-4H-1,2,4-triazol-3-yl}methyl)-2-furamide
Metabolite276	N~2~-[(2R)-3-{5-[(4-Carbamimidoylphenoxy)carbonyl]-2-furyl}-2-methylpropanoyl]-L-asparagine
Metabolite277	(2S)-{[(2R)-2-Amino-3-(benzylsulfanyl)-3-methylbutanoyl]amino}[(2R,3S,4R)-5-(2,4-dioxo-3,4-dihydro-1(2H)-pyrimidinyl)-3,4-dihydroxytetrahydro-2-furanyl]acetic acid
Metabolite278	(3S,4R)-3,4,5-Trihydroxy-4'-oxo-3',4,4',5-tetrahydro-2'H,3H-spiro[furan-2,1'-naphthalene]-6'-carboxylic acid
Metabolite279	Hexyl (2E)-2-[(4-methyl-5-oxido-1,2,5-oxadiazol-3-yl)methylene]hydrazinecarboxylate
Metabolite280	2-(Hexopyranosyloxy)ethyl hexanoate
Metabolite281	trans-Aconitic acid
Metabolite282	N-(3,4-Dichlorophenyl)-6-methoxy-7-{[4-(2-methoxyethyl)-1,4-oxazepan-2-yl]methoxy}-4-quinazolinamine
Metabolite283	4,4'-[(5-Bromo-3-chloro-2-hydroxyphenyl)methylene]bis(5-methyl-2-phenyl-1,2-dihydro-3H-pyrazol-3-one)
Metabolite284	alpha-Ketoglutaric acid
Metabolite285	3-(Carboxymethyl)-2,6-dihydroxy-4-methoxy-5-(3-methyl-2-buten-1-yl)phenyl hexopyranosiduronic acid
Metabolite286	(3S,4S)-5-Cyclohexyl-3-hydroxy-N-[3-(1H-imidazol-1-yl)propyl]-4-{[(2S)-4-methyl-2-{[(2S)-2-{[3-(3-oxo-2,3-dihydro-4H-pyrido[4,3-b][1,4]oxazin-4-yl)propanoyl]amino}-3-phenylpropanoyl]amino}pentanoyl]amino}pentanamide
Metabolite287	N-(4-Methoxybenzyl)-4-{4-[(2-methyl-5-nitrophenyl)sulfonyl]-1-piperazinyl}-4-oxobutanamide
Metabolite288	2-(Benzyloxy)ethyl {(1R,2R,6S,6aS,11bS,11cS)-6a-(allyloxy)-4-(ethoxyimino)-1,2-bis(4-hydroxybutyl)-10-[(6-methyl-2-pyridinyl)methoxy]-1,2,4,5,6,6a,11b,11c-octahydrobenzo[kl]xanthen-6-yl}[2-(2-hydroxyethoxy)ethyl]carbamate
Metabolite289	(2S,3R,4R,5R,6S)-2-{[(2R,3S,4R,5R,6S)-6-{[(2R,3R,4S,5S,6R)-4,5-dihydroxy-6-(hydroxymethyl)-2-[(1'S,2S,4'S,5S,7'R,9'S,13'R,16'S)-5,7',9',13'-tetramethyl-5-({[(2R,3R,4S,5S,6R)-3,4,5-trihydroxy-6-(hydroxymethyl)oxan-2-yl]oxy}methyl)-5'-oxaspiro[oxolane-2,6'-pentacyclo-icosan]-18'-eneoxy]oxan-3-yl]oxy}-4,5-dihydroxy-2-(hydroxymethyl)oxan-3-yl]oxy}-6-methyloxane-3,4,5-triol
Metabolite290	Glomeratose A
Metabolite291	3-(2,3-Dihydroxybutyl)-7-hydroxy-2-benzofuran-1(3H)-one

Metabolite292	(3beta,25S)-3-{[6-Deoxy-alpha-L-mannopyranosyl-(1->2)-[6-deoxy-alpha-L-mannopyranosyl-(1->4)]-beta-D-glucopyranosyl]oxy}-2-oxofurosta-5,20(22)-dien-26-yl beta-D-glucopyranoside
Metabolite293	(7R,7aS,10aR,11aR)-7a,10a-Dichloro-7-[2-hydroxy-5-(trifluoromethoxy)phenyl]-9-methyl-2-phenyl-7a,10a,11,11a-tetrahydro-1H,5H-pyrrolo[3,4-g][1,2,4]triazolo[1,2-a]cinnoline-1,3,8,10(2H,7H,9H)-tetrone
Metabolite294	(3beta,14beta,17alpha,20R)-3-[(6-Deoxy-3-O-methylhexopyranosyl)oxy]-14-hydroxypregnan-20-yl hexopyranoside
Metabolite295	DL-Tryptophan
Metabolite296	2-(2,6-Dichlorophenyl)-1-[4-(2-thienylsulfonyl)-1-piperazinyl]ethanone
Metabolite297	2,2'-Methylenebis(4-methyl-6-tert-butylphenol)
Metabolite298	Tetraethyl 1,6-diacetamido-1,1,6,6-hexanetetracarboxylate
Metabolite299	Silandrin
Metabolite300	6-[(Ethoxycarbonyl)amino]-3-oxo-2,8-dioxabicyclo[3.2.1]oct-7-yl acetate
Metabolite301	4-(1-{[(2S,3S,4R)-3,4-Dihydroxy-2-pyrrolidinyl]methyl}-1H-1,2,3-triazol-4-yl)butanoic acid
Metabolite302	Dimethyl (6,7,8-trimethoxy-4-oxo-1,2,3,4-tetrahydroquinazoline-2,2-diyl)biscarbamate
Metabolite303	(3S)-5-{[(2R,3S,4S,5R,6R)-6-{[(2R,3S,4S,5R,6S)-6-({[(4aR,5R,6aS,6bR,8aR,9S,10S,12aR,12bR,14bS)-5,10-Dihydroxy-2,2,6a,6b,9,12a-hexamethyl-9-({[(2S,3R,4S,5S,6R)-3,4,5-trihydroxy-6-(hydroxymethyl)tetrahydro-2H-pyran-2-yl]oxy}carbonyl)-1,3,4,5,6,6a,6b,7,8,8a,9,10,11,12,12a,12b,13,14b-octadecahydro-4a(2H)-picenyl]carbonyl}oxy)-3,4,5-trihydroxytetrahydro-2H-pyran-2-yl]methoxy}-3,4-dihydroxy-5-{[(2S,3R,4S,5S,6R)-3,4,5-trihydroxy-6-(hydroxymethyl)t
Metabolite304	(6R)-5-Acetamido-4-amino-2,6-anhydro-6-(3-butyl-1,2,4-oxadiazol-5-yl)-3,4,5-trideoxy-L-threo-hex-2-enonic acid
Metabolite305	3-Coumaric acid
Metabolite306	D-Xylonic acid
Metabolite307	MFCD02329635
Metabolite308	N-{1-[(4-Morpholinylcarbonyl)amino]ethyl}-alpha-asparagine
Metabolite309	Dimethyl 1-(3,4-dimethoxybenzyl)-4-(3-phenoxyphenyl)-1,4-dihydro-3,5-pyridinedicarboxylate
Metabolite310	Isorhamnetin 7-alpha-D-Glucosamine
Metabolite311	N-[(2-Chlorobenzyl)(cyclohexyl)carbamothioyl]glycine
Metabolite312	3,6-Bis-O-[(2E)-3-(4-hydroxy-3-methoxyphenyl)-2-propenoyl]-beta-D-fructofuranosyl 2-O-acetyl-alpha-D-glucopyranoside
Metabolite313	L-Tyrosine
Metabolite314	2-[[7-(1,4-dioxa-8-azaspiro[4.5]dec-8-yl)-4-nitro-2,1,3-benzoxadiazol-5-yl](methyl)amino]ethanol
Metabolite315	(1S,4R)-4-Hydroxy-2,2-dimethyl-4-{5-[3-(1H-pyrazol-4-yl)-5-{[4-(trifluoromethyl)-2-pyrimidinyl]amino}phenyl]-1,3-thiazol-2-yl}cyclohexanecarboxylic acid
Metabolite316	5-(acetylamino)-2,6-anhydro-3,4,5-trideoxy-4-[4-(1-hydroxypropyl)-1H-1,2,3-triazol-1-yl]-D-glycero-D-galacto-non-2-enonic acid
Metabolite317	(1'R,2S,4'S,5S,6R,8'R,10'E,12'S,13'S,14'E,16'E,20'R,21'R,24'S)-6-[(2R)-2-Butanyl]-21',24'-dihydroxy-5,11',13',22'-tetramethyl-2'-oxo-5,6-dihydrospiro[pyran-2,6'-[3,7,19]trioxatetracyclo[15.6.1.1~4,8~.0~20,24~]pentacosa[10,14,16,22]tetraen]-

	12'-yl 4-O-[4-S-(2-acetamidoethyl)-2,6-dideoxy-3-O-methyl-4-thio-alpha-L-lyxo-hexopyranosyl]-2,6-dideoxy-3-O-methyl-alpha-L-arabino-hexopyranoside
Metabolite318	N-[(1R,2R,6S,6aS,11bS,11cS)-6a-(Allyloxy)-4-[(benzyloxy)imino]-10-[(2-fluorobenzyl)oxy]-1,2-bis(4-hydroxybutyl)-1,2,4,5,6,6a,11b,11c-octahydrobenzo[kl]xanthen-6-yl]-3-cyclopentyl-N-(1-naphthylmethyl)propanamide
Metabolite319	N-[(5S)-5-{[(2S)-2-Acetamido-3-(2-naphthyl)propanoyl]amino}-6-({1-[(2S)-2-carbamoyl-1-pyrrolidinyl]-6-(isopropylamino)-1-oxo-2-hexanyl}amino)-6-oxohexyl]-2-pyridinecarboxamide
Metabolite320	4-Methylene-2-oxoglutarate
Metabolite321	Protogracillin
Metabolite322	(2S)-3-Amino-N-[(2S,3S)-2-amino-3-hydroxy-3-(4-nitrophenyl)propyl]-2-hydroxypropanamide
Metabolite323	1-(D-Ribofuranosyl)-1,3-diazepane-2,5-dione
Metabolite324	2-(6-Amino-1,3-dimethyl-2,4-dioxo-1,2,3,4-tetrahydro-5-pyrimidinyl)-2-oxoethyl 4-methyl-3-(1-piperidinylsulfonyl)benzoate
Metabolite325	δ-Gluconic acid δ-lactone
Metabolite326	4-[4-(beta-Alanylamino)benzyl]-5-isopropyl-1H-pyrazol-3-yl beta-D-glucopyranoside
Metabolite327	Ethyl (2,6-dinitro-1H-benzimidazol-1-yl)acetate
Metabolite328	L-Histidine
Metabolite329	(1R,18R,20R,24S,27S)-24-Cyclohexyl-N-[(1R,2S)-1-{[(1-methylcyclopropyl)sulfonyl]carbamoyl}-2-vinylcyclopropyl]-11-[3-(4-methyl-1-piperazinyl)propoxy]-22,25-dioxo-2,21-dioxa-4,23,26-triazapentacyclo[24.2.1.0~3,12~.0~5,10~.0~18,20~]nonacosa-3,5,7,9,11-pentaene-27-carboxamide
Metabolite330	(2R,3S,5R)-5-(4-Amino-2-oxo-1(2H)-pyrimidinyl)-2-(hydroxymethyl)tetrahydro-3-furanyl [(2R,3S,5R)-5-(6-amino-9H-purin-9-yl)-3-hydroxytetrahydro-2-furanyl]methyl hydrogen phosphate
Metabolite331	Dithiocyano methane
Metabolite332	3-O-beta-D-galactosyl-sn-glycerol
Metabolite333	Protirelin
Metabolite334	5-Butoxy-2-[(4-{[(2,4-diamino-6-pteridinyl)methyl](methyl)amino}benzoyl)amino]-5-oxopentanoic acid
Metabolite335	N-{[(2-Methyl-2-propanyl)oxy]carbonyl}-L-phenylalanyl-L-phenylalanyl-L-allothreonyl-L-leucyl-L-alpha-aspartyl-L-alanyl-L-alpha-aspartyl-L-phenylalanine
Metabolite336	2-{4-[5-(4-Methoxyphenyl)thieno[2,3-d]pyrimidin-4-yl]-1-piperazinyl}phenol
Metabolite337	muramic acid
Metabolite338	N-[(2R)-2-Acetamido-2-(10,11-dihydro-5H-dibenzo[a,d][7]annulen-5-yl)acetyl]-L-leucyl-L-alpha-aspartyl-L-isoleucyl-L-isoleucyl-L-tryptophan
Metabolite339	(3beta,15alpha,16alpha,21beta,22alpha)-15,16,21,22,24,28-Hexahydroxyolean-12-en-3-yl alpha-L-arabinofuranosyl-(1->3)-[beta-D-glucopyranosyl-(1->2)]-beta-D-glucopyranosiduronic acid
Metabolite340	4-Undecylbenzenesulfonic acid
Metabolite341	2-(2,4,6-Trichlorophenoxy)ethanol
Metabolite342	1-({[(2-Hydroxyethyl)disulfanyl]carbonyl}oxy)decane
Metabolite343	1,3-Benzothiazol-2-ylmethyl 1-(2-thienylsulfonyl)-4-piperidinecarboxylate
Metabolite344	2-Hydroxycinnamic acid
Metabolite345	Deoxynivalenol 3-glucuronide

Metabolite346	L-Glutathione (reduced)
Metabolite347	(2S)-2-[(3-Carboxypropanoyl)amino]hexanedioic acid
Metabolite348	Uridine 5'-diphosphogalactose
Metabolite349	Epirizole
Metabolite350	6-O-beta-D-Glucopyranosyl-1-O-[(2alpha,3beta)-2,20,23-trihydroxy-28-oxo-3-{[3-O-(beta-D-xylopyranosyl)-alpha-L-arabinopyranosyl]oxy}urs-12-en-28-yl]-beta-D-glucopyranose
Metabolite351	THREONIC ACID, L-
Metabolite352	2-(4-Aminopyrrolo[2,1-f][1,2,4]triazin-7-yl)-2,5-anhydro-1-deoxy-3-C-methyl-D-altritol
Metabolite353	3-Hydroxy-3-methylglutaric acid
Metabolite354	2-{2-[8,9-Di(2-furyl)furo[3,2-e][1,2,4]triazolo[1,5-c]pyrimidin-2-yl]ethyl}-1H-isoindole-1,3(2H)-dione
Metabolite355	N-Acetyl-L-seryl-L-threonyl-D-prolyl-L-proline
Metabolite356	Oxyisocyclointegrin
Metabolite357	(5S)-N-[(2S,3R)-3-Hydroxy-4-{isobutyl[(4-methoxyphenyl)sulfonyl]amino}-1-phenyl-2-butanyl]-2-oxo-3-[3-(trifluoromethyl)phenyl]-1,3-oxazolidine-5-carboxamide
Metabolite358	[(2,2-Dimethylpropanoyl)oxy]methyl (2R,3R,5S,6S)-6-{[(2R)-2-{[(4-ethyl-2,3-dioxo-1-piperazinyl)carbonyl]amino}-2-phenylacetyl]amino}-4-hydroxy-3-methyl-7-oxo-1-azabicyclo[3.2.0]heptane-2-carboxylate
Metabolite359	2-OXOADIPATE
Metabolite360	N-[(2S,3R,4S)-1-Cyclohexyl-3,4-dihydroxy-6-(2-pyridinyl)-2-hexanyl]-Nalpha-{(2S)-2-[(methyl{2-[methyl(4-morpholinylcarbonyl)amino]ethyl}carbamoyl)oxy]-3-phenylpropanoyl}-L-histidinamide
Metabolite361	1-(2-Deoxypentofuranosyl)-5-methyl-2,4(1H,3H)-pyrimidinedione
Metabolite362	N-[(2E)-3-(4-Hydroxyphenyl)-2-propenoyl]-L-alanyl-D-alpha-glutaminyl-N~6~-[3-({(2R,3S)-1-{[(2alpha,5beta,7beta,10beta,13alpha)-4-acetoxy-2-(benzoyloxy)-1,7,10-trihydroxy-9-oxo-5,20-epoxytax-11-en-13-yl]oxy}-3-[(2,2-dimethylpropanoyl)amino]-1-oxo-3-phenyl-2-propanyl}oxy)propanoyl]-L-lysine
Metabolite363	Rosaramicin propionate
Metabolite364	(1R,2R,4S)-2-Hydroxy-4-(2-hydroxy-2-propanyl)-1-methylcyclohexyl beta-L-glucopyranoside
Metabolite365	N-[3-({2-[(2-Methoxyethyl)amino]-6-phenylthieno[3,2-d]pyrimidin-4-yl}oxy)phenyl]acetamide
Metabolite366	(3R)-6-Cyclohexyl-N-hydroxy-3-[3-(4-morpholinylcarbonyl)-1,2,4-oxadiazol-5-yl]hexanamide
Metabolite367	2-deoxyglucose
Metabolite368	[(2S,3S,4R,5R)-4-hydroxy-2,5-bis(hydroxymethyl)-2-[(2R,3R,4S,5S,6R)-3,4,5-trihydroxy-6-(hydroxymethyl)oxan-2-yl]oxyoxolan-3-yl] (E)-3-(4-hydroxy-3-methoxyphenyl)prop-2-enoate
Metabolite369	[(4E)-4-(4-Chlorobenzylidene)-2-(hydroxymethyl)-5-oxotetrahydro-2-furanyl]methyl 4-chlorobenzoate
Metabolite370	Methyl 7,12-dioxo-5-{[4-(trifluoromethyl)benzyl]oxy}-7,12-dihydrodinaphtho[1,2-b:2',3'-d]furan-6-carboxylate
Metabolite371	1,28-Dihydroxy-26-oxo-22,26-epoxyergosta-5,24-dien-3-yl 6-O-hexopyranosylhexopyranoside
Metabolite372	5-(3,4-Dihydroxy-5-nitrophenyl)pentanoic acid

Metabolite373	[3-Chloro-5-(2-thienyl)-7-(trifluoromethyl)pyrazolo[1,5-a]pyrimidin-2-yl](3,4-dihydro-1(2H)-quinolinyl)methanone
Metabolite374	N-SUCCINYL ASPARTIC ACID
Metabolite375	kanamycin A 3'-phosphate
Metabolite376	3-(3,5-Dioxo-2,3,4,5-tetrahydro-1,2,4-triazin-6-yl)-N-(3-nitrophenyl)propanamide
Metabolite377	Methyl 3-[(4-propylphenyl)amino]-1-(2,3,5-tri-O-acetyl-beta-D-ribofuranosyl)-1H-1,2,4-triazole-5-carboxylate
Metabolite378	1-(4-Fluorobenzoyl)-2,2,4-trimethyl-1,2-dihydro-6-quinolinyl 4-nitrobenzoate
Metabolite379	(2S,3S,4R,5R,6R)-6-[[(3R,6aR,6bS,8R,8aR,14bR)-8-hydroxy-4,4,6a,6b,11,11,14b-heptamethyl-8a-[(2S,3R,4S,5S,6R)-3,4,5-trihydroxy-6-(hydroxymethyl)oxan-2-yl]oxycarbonyl-1,2,3,4a,5,6,7,8,9,10,12,12a,14,14a-tetradecahydropicen-3-yl]oxy]-4,5-dihydroxy-3-[(2S,3R,4S,5R)-3,4,5-trihydroxyoxan-2-yl]oxyoxane-2-carboxylic acid
Metabolite380	Lumota
Metabolite381	(2S,3R,4R,5R,6S)-2-{[(2R,3R,4S,5R,6R)-4,5-dihydroxy-6-methyl-2-[(2R,4'S,7'S,8'R,9'S,13'R,14'R,16'R)-7',9',13'-trimethyl-5-methylidene-16'-{[(2R,3R,4R,5R,6S)-3,4,5-trihydroxy-6-methyloxan-2-yl]oxy}-5'-oxaspiro[oxane-2,6'-pentacyclo-icosan]-18'-eneoxy]oxan-3-yl]oxy}-6-methyloxane-3,4,5-triol
Metabolite382	(2R,3S,4S,5R,6R)-5-[(2S,3R,4R)-3,4-dihydroxy-4-(hydroxymethyl)oxolan-2-yl]oxy-2-(hydroxymethyl)-6-[2-(4-hydroxyphenyl)ethoxy]oxane-3,4-diol
Metabolite383	5-(5-Bromo-2-thienyl)-7-[2-(difluoromethoxy)phenyl]-1,5,6,7-tetrahydro[1,2,4]triazolo[1,5-a]pyrimidine
Metabolite384	5-(1,2-Dithiolan-3-yl)-N-(2-propyn-1-yl)pentanamide
Metabolite385	263CU738ZY
Metabolite386	9,10-Dihydro-10- (3,4-dihydroxyphenyl) -pyrano [2,3-h] catechin-8-one
Metabolite387	2-[4-(2-Hydroxyethyl)-1-piperazinyl]ethyl (2Z)-4-[(1S,2S,8R,17S,19R)-12-hydroxy-8,21,21-trimethyl-5-(3-methyl-2-buten-1-yl)-8-(4-methyl-3-penten-1-yl)-14,18-dioxo-3,7,20-trioxahexacyclo[15.4.1.0~2,15~.0~2,19~.0~4,13~.0~6,11~]docosa-4(13),5,9,11,15-pentaen-19-yl]-2-methyl-2-butenoate
Metabolite388	(1R,3S,4S,7S,9S,10S,12S,13S,15Z,17E,19R,21R,23S)-23-[(3,4-Di-O-butyryl-6-deoxy-2-O-methyl-alpha-L-talopyranosyl)oxy]-4-hydroxy-12,19-dimethyl-5-oxo-6,25,26-trioxatetracyclo[19.3.1.1~4,7~.1~10,13~]heptacosa-15,17-diene-3,9-diyl diacetate
Metabolite389	3-Allyl-2-hydroxybenzoic acid
Metabolite390	5-[2,4-Dihydroxy-6-(4-nitrophenoxy)phenyl]-N-[3-(dimethylamino)propyl]-1,2-oxazole-3-carboxamide
Metabolite391	2-[(2E)-3-(4-Bromophenyl)-2-propen-1-ylidene]-1,3-cyclopentanedione
Metabolite392	Adenylthiomethylpentose
Metabolite393	2-(Benzyloxy)ethyl [(1R,2R,6S,6aS,11bS,11cS)-6a-(allyloxy)-1,2-bis(4-hydroxybutyl)-4-(methoxyimino)-10-(2-naphthyloxy)-1,2,4,5,6,6a,11b,11c-octahydrobenzo[kl]xanthen-6-yl](1-naphthylmethyl)carbamate
Metabolite394	N-(1,5-Dimethyl-3-oxo-2-phenyl-2,3-dihydro-1H-pyrazol-4-yl)-1,3-dimethyl-2-oxo-2,3-dihydro-1H-benzimidazole-5-sulfonamide
Metabolite395	N-[(2S,3S)-2-Amino-3-hydroxy-3-(4-nitrophenyl)propyl]-L-serinamide
Metabolite396	N~2~-[(3,4-Dichlorophenyl)carbamoyl]-N-hydroxy-N~2~-(2-nitrobenzyl)alaninamide
Metabolite397	N-({5-[(3R)-6-Cyclohexyl-1-(hydroxyamino)-1-oxo-3-hexanyl]-1,2,4-oxadiazol-3-yl}carbonyl)glycine

Metabolite398	8-Amino-1-methyl-2-(methylsulfanyl)-9-pentopyranosyl-1,9-dihydro-6H-purin-6-one
Metabolite399	(1E)-1-(5-Bromo-1,3-benzoxazol-2-yl)-N-hydroxy-2-phenylethanimine
Metabolite400	Kaplanin
Metabolite401	1,4-Bis(5-ethylsulfonylbenzoxazole-2-yl)naphthalene
Metabolite402	(E)-[2,6-Dimethyl-4-{2-[(4-methyl-5-oxido-1,2,5-oxadiazol-3-yl)methoxy]phenyl}-5-nitro-3(4H)-pyridinylidene](methoxy)methanol
Metabolite403	(3E)-1,1,1-Trifluoro-4-phenyl-3-buten-2-one
Metabolite404	2-(Benzyloxy)ethyl [(1R,2R,6S,6aS,11bS,11cS)-6a-(allyloxy)-4-[(benzyloxy)imino]-10-(4-biphenylyloxy)-1,2-bis(4-hydroxybutyl)-1,2,4,5,6,6a,11b,11c-octahydrobenzo[kl]xanthen-6-yl]propylcarbamate
Metabolite405	1-Acetyl-D-prolyl-L-seryl-D-prolyl-L-serine
Metabolite406	(4R,5S)-4-(6-Amino-9H-purin-9-yl)-5-hydroxy-2-(hydroxymethyl)-2-cyclopenten-1-one
Metabolite407	Arabinosylhypoxanthine
Metabolite408	(2S)-[(2R,3S,4R,5R)-5-(2,4-Dioxo-3,4-dihydro-1(2H)-pyrimidinyl)-3,4-dihydroxytetrahydro-2-furanyl]{[2-(methylamino)hexanoyl]amino}acetic acid
Metabolite409	(3R,4S,5S,6R,7R,9R,11S,12R,13R,14R)-6-{[(2S,3R,4S,6R)-4-(Dimethylamino)-3-hydroxy-6-methyltetrahydro-2H-pyran-2-yl]oxy}-14-ethyl-13-hydroxy-7,12-dimethoxy-3,5,7,9,11,13-hexamethyl-2,10-dioxooxacyclotetradecan-4-yl [2-(2-chlorophenyl)ethyl]carbamate
Metabolite410	nocardicin A
Metabolite411	4-[4-(4-Hydroxy-3-methoxyphenyl)tetrahydro-1H,3H-furo[3,4-c]furan-1-yl]-2-methoxyphenyl hexopyranoside
Metabolite412	Cyclo(3-amino-3-oxo-L-alanyl-L-prolyl-L-valyl-L-valyl-L-histidyl-L-phenylalanyl-L-phenylalanyl-L-tyrosyl-L-asparaginyl-L-isoleucyl-L-valyl-L-threonyl-L-alanyl-L-arginyl-L-threonyl-L-prolyl-L-alpha-glutamyl)
Metabolite413	Robinetinidol 3-O-gallate
Metabolite414	Trimethyl (1R,4E,5R,7R,8R,9R)-1-hydroxy-4-[hydroxy(methoxy)methylene]-7-(4-nitrophenyl)-3-oxobicyclo[3.3.1]nonane-2,8,9-tricarboxylate
Metabolite415	(3R,4S,4aS,9bS)-8-Formyl-4-hydroxy-N-(2-hydroxyethyl)-6-methoxy-3-{[(2S)-tetrahydro-2-furanylmethyl][4-(trifluoromethyl)benzoyl]amino}-3,4,4a,9b-tetrahydrodibenzo[b,d]furan-1-carboxamide
Metabolite416	N-[4-(2-{[4-(2-Hydrazino-2-oxoethyl)phenyl]amino}-2-oxoethyl)phenyl]-9-(D-ribofuranosyl)-9H-purin-6-amine
Metabolite417	3-Ethyl-2-methyl-7-phenyl-N-[2-(trifluoromethyl)phenyl]-4,7-dihydrothieno[2,3-c]pyridine-6(5H)-carboxamide
Metabolite418	Propentofylline
Metabolite419	(Z)-3-[4-methoxy-2-[(2S,3R,4S,5S,6R)-3,4,5-trihydroxy-6-(hydroxymethyl)oxan-2-yl]oxyphenyl]prop-2-enoic acid
Metabolite420	S(8-8)S hexoside
Metabolite421	L-Asparaginyl-L-methionyl-L-valyl-L-prolyl-L-phenylalanyl-L-phenylalanyl-L-prolyl-L-prolyl-L-valine
Metabolite422	2-[(1R,2S,4S)-2-Hydroxy-1,4-bis(hydroxymethyl)cyclohexyl]ethyl beta-D-glucopyranoside
Metabolite423	(3beta,16alpha)-16,28-Dihydroxy-23,28-dioxoolean-12-en-3-yl beta-D-galactopyranosyl-(1->2)-[beta-D-glucopyranosyl-(1->3)]-beta-D-glucopyranosiduronic acid
Metabolite424	β-D-Glucopyranuronic acid
Metabolite425	GLOBOSUMONE B

Metabolite426	2-Amino-4-(6-bromo-1,3-benzodioxol-5-yl)-5-oxo-5,6,7,8-tetrahydro-4H-chromene-3-carbonitrile
Metabolite427	Acefylline
Metabolite428	(3beta,15alpha,16alpha,21beta,22alpha)-15,16,21,22,28-Pentahydroxyolean-12-en-3-yl beta-D-galactopyranosyl-(1->2)-[beta-D-xylopyranosyl-(1->2)-alpha-L-arabinopyranosyl-(1->3)]-beta-D-glucopyranosiduronic acid
Metabolite429	Glycyl-L-prolyl-L-prolyl-L-isoleucyl-L-valyl-L-prolyl-L-tyrosyl-L-phenylalanine
Metabolite430	3,5-dihydroxydecanoic acid
Metabolite431	4-{2-[(4-Acetamidophenyl)sulfonyl]hydrazino}-N-benzyl-4-oxobutanamide
Metabolite432	(1aR,2E,4aR,6S,7S,7aR,8R,9S,11aS)-4a-Hydroxy-1,1,3,6-tetramethyl-4-oxo-1,1a,4,4a,5,6,7,7a,8,10,11,11a-dodecahydrospiro[cyclopenta[a]cyclopropa[f][11]annulene-9,2'-oxirane]-7,8-diyl di(2-naphthoate)
Metabolite433	[(1aS,1bS,5S,5aS,6S)-6-hydroxy-6-(hydroxymethyl)-2-[[(2R,3R,4S,5S,6R)-3,4,5-trihydroxy-6-(hydroxymethyl)oxan-2-yl]oxymethyl]-1b,5,5a,6a-tetrahydro-1aH-oxireno[3,4]cyclopenta[1,3-d]pyran-5-yl] 3-methylbutanoate
Metabolite434	Glibenclamide
Metabolite435	3-Hydroxy-3-(methoxycarbonyl)pentanedioic acid
Metabolite436	[6-[3,4-dihydroxy-2,5-bis(hydroxymethyl)oxolan-2-yl]oxy-3,4,5-trihydroxyoxan-2-yl]methyl (E)-3-(4-hydroxy-3-methoxyphenyl)prop-2-enoate
Metabolite437	5-[(2-Amino-6-hydroxy-1H-purin-1-yl)methyl]-5-methyl-3-methylenedihydro-2(3H)-furanone
Metabolite438	(3Z)-3-[Hydroxy(2-methoxyethoxy)methylene]-2-methyl-4-(3-nitrophenyl)-4H-pyrimido[1,6-a]pyrimidine-6,8(3H,7H)-dione
Metabolite439	NCGC00385663-01_C33H40O18_6-O-beta-D-Glucopyranosyl-1-O-[(2E)-3-(4-hydroxy-3,5-dimethoxyphenyl)-2-propenoyl]-2-O-[(2E)-3-(4-hydroxy-3-methoxyphenyl)-2-propenoyl]-beta-D-glucopyranose
Metabolite440	3,4,5-trihydroxy-6-(hydroxymethyl)oxan-2-yl (2E,6R)-6-[(1R,3R,6S,8R,12S,15R,16R)-13,17-dihydroxy-7,7,12,16-tetramethyl-6-{[(2R)-3,4,5-trihydroxy-6-{[(3,4,5-trihydroxyoxan-2-yl)oxy]methyl}oxan-2-yl]oxy}pentacyclo-octadecan-15-yl]-2-methylhept-2-enoate
Metabolite441	Medicagenic acid 3-O-triglucoside
Metabolite442	(1S,2S,4S,5R,6S,7S,9R,12R)-6-(Acetoxymethyl)-2,12-dihydroxy-2,10,10-trimethyl-8-oxo-11-oxatricyclo[7.2.1.0~1,6~]dodecane-4,5,7-triyl triacetate
Metabolite443	Bis(4-methoxyphenyl) ({2-[(6-amino-9H-purin-9-yl)oxy]ethoxy}methyl)phosphonate
Metabolite444	N-Benzoylglycyl-L-valyl-L-leucyl-N~6~-[(3S)-3-carboxy-3-(phenylsulfanyl)propanoyl]-L-lysyl-L-alpha-glutamyl-L-tyrosylglycyl-L-valinamide
Metabolite445	5-Oxo-L-prolyl-L-alpha-glutamyl-L-prolinamide
Metabolite446	(3beta,16alpha,21beta)-3-{[alpha-L-Arabinopyranosyl-(1->6)-[beta-D-glucopyranosyl-(1->2)]-beta-D-glucopyranosyl]oxy}-16,21-dihydroxyolean-12-en-28-oic acid
Metabolite447	Methyl 6-amino-4-(2-bromophenyl)-5-cyano-2-(methoxymethyl)-4H-pyran-3-carboxylate
Metabolite448	NCGC00385695-01_C21H28O13_alpha-D-Glucopyranoside, beta-L-fructofuranosyl 6-O-[(2E)-3-(4-hydroxyphenyl)-1-oxo-2-propen-1-yl]-
Metabolite449	(3beta,21beta,22beta)-22-{[2-O-(beta-D-Allopyranosyl)-alpha-L-lyxopyranosyl]oxy}-21,24-dihydroxyolean-12-en-3-yl 2-O-beta-D-allopyranosyl-beta-D-glucopyranosiduronic acid
Metabolite450	1-[(Benzyloxy)methoxy]-2,3-diphenyl-7-indolizinecarbonitrile

Metabolite451	2-Chloroethyl [(1R,2R,6S,6aS,11bS,11cS)-6a-(allyloxy)-10-[(2-fluorobenzyl)oxy]-1,2-bis(4-hydroxybutyl)-4-{[(2-methyl-2-propanyl)oxy]imino}-1,2,4,5,6,6a,11b,11c-octahydrobenzo[kl]xanthen-6-yl]propylcarbamate
Metabolite452	7-Hexyl-3-methyl-8-[(2-oxo-2-phenylethyl)sulfanyl]-3,7-dihydro-1H-purine-2,6-dione
Metabolite453	ACVA
Metabolite454	D-Galactose
Metabolite455	Tetrahydro-2-furanylmethyl 4-(6-chloro-4-oxo-4H-chromen-3-yl)-2-methyl-5-oxo-1,4,5,6,7,8-hexahydro-3-quinolinecarboxylate
Metabolite456	MCA
Metabolite457	KN-62
Metabolite458	1,3,4,5-Tetrahydroxycyclohexanecarboxylic acid
Metabolite459	1-{(2R,3R,4S,5S)-3,4-Dihydroxy-5-[(3S)-5-oxo-1,2-dithiolan-3-yl]tetrahydro-2-furanyl}-2,4(1H,3H)-pyrimidinedione
Metabolite460	6-Deoxy-beta-L-mannopyranosyl-(1->4)-beta-L-glucopyranosyl-(1->6)-1-O-[(3alpha,20S)-3,20,23,29-tetrahydroxy-23,28-dioxolupan-28-yl]-beta-L-glucopyranose
Metabolite461	2-Phenoxyethyl 2-methyl-4-(3-nitrophenyl)-5-oxo-7-(2-thienyl)-1,4,5,6,7,8-hexahydro-3-quinolinecarboxylate
Metabolite462	1-(4-Chloro-2,5-dimethoxyphenyl)-3-[2-(1H-indol-3-yl)ethyl]thiourea
Metabolite463	2-isopropylmaleic acid
Metabolite464	(4S)-5-[4-(Ethoxycarbonyl)-1-piperazinyl]-4-({[5-(2-ethoxy-2-oxoethoxy)-1-phenyl-1H-pyrazol-3-yl]carbonyl}amino)-5-oxopentanoic acid
Metabolite465	5-Nitro-1-(4-nitrophenyl)dihydro-2,4(1H,3H)-pyrimidinedione
Metabolite466	Pinoresinol diglucoside
Metabolite467	N-[1-(5-{[2-(4-Bromophenyl)-2-oxoethyl]sulfanyl}-4-ethyl-4H-1,2,4-triazol-3-yl)ethyl]-2-(4-methoxyphenyl)acetamide
Metabolite468	Dodecyl sulfate
Metabolite469	Dicoumaroyl Spermidine
Metabolite470	[4,5,8-trihydroxy-5-(hydroxymethyl)-3-(3-methoxy-3-oxoprop-1-en-2-yl)-8a-methyl-1,2,3,4,4a,6,7,8-octahydronaphthalen-2-yl] 3,4-dihydroxy-2-methylidenebutanoate
Metabolite471	Sibiricose A1
Metabolite472	(2S,3R,4R,5R,6S)-2-{[(2R,3S,4S,5R,6R)-5-{[(2S,3R,4S,5R,6R)-3,5-dihydroxy-6-(hydroxymethyl)-4-{[(2S,3R,4S,5S,6R)-3,4,5-trihydroxy-6-(hydroxymethyl)oxan-2-yl]oxy}oxan-2-yl]oxy}-4-hydroxy-2-(hydroxymethyl)-6-[(1'S,2S,4'S,5S,7'S,8'R,9'S,13'R,16'S)-5,7',9',13'-tetramethyl-5-({[(2R,3R,4S,5S,6R)-3,4,5-trihydroxy-6-(hydroxymethyl)oxan-2-yl]oxy}methyl)-5'-oxaspiro[oxolane-2,6'-pentacyclo-icosan]-18'-eneoxy]oxan-3-yl]oxy}-6-methyloxane-3,4,5-triol
Metabolite473	Ferulic acid
Metabolite474	Disaccharides ((2Methyl-Hex)-Pen)
Metabolite475	Benzoic acid + 1O, O-Hex
Metabolite476	4-Dodecylbenzenesulfonic acid
Metabolite477	L-Glutathione oxidized
Metabolite478	Quercetin-3β-D-glucoside
Metabolite479	Coumaroyl Hexoside (isomer of 690, 692)
Metabolite480	9(Z),11(E)-Conjugated linoleic acid

Metabolite481	Orcinol gentiobioside
Metabolite482	Pimelic acid
Metabolite483	Benzoic acid + 1O, 1MeO, O-Hex
Metabolite484	[(2R,3S,4S,5R,6R)-3,4,5-trihydroxy-6-[(2S,3S,4R,5R)-4-hydroxy-3-[(E)-3-(4-hydroxy-3,5-dimethoxyphenyl)prop-2-enoyl]oxy-2,5-bis(hydroxymethyl)oxolan-2-yl]oxyoxan-2-yl]methyl (E)-3-(4-hydroxy-3,5-dimethoxyphenyl)prop-2-enoate
Metabolite485	1,6-Bis-O-[(2E)-3-(4-hydroxyphenyl)-2-propenoyl]-β-D-glucopyranose
Metabolite486	Astragalin
Metabolite487	4-Acetyl-3-hydroxy-5-methylphenyl β-D-glucopyranoside
Metabolite488	N-Acetyl-DL-tryptophan
Metabolite489	Isorhamnetin-3-O-rutinoside
Metabolite490	Asperulosidic acid
Metabolite491	Quinic acid
Metabolite492	Propyl gallate
Metabolite493	7-O-Methylquercetin-3-O-galactoside-6''-rhamnoside
Metabolite494	Docosahexaenoic acid
Metabolite495	Rutin
Metabolite496	Lariciresinol 4-O-glucoside
Metabolite497	Benzoic acid + 2O, O-Hex
Metabolite498	Geniposide
Metabolite499	Abietic acid
Metabolite500	N-{2-Chloro-4-[({3-[4-(2-chloro-6-fluorobenzyl)piperazino]propyl}amino)sulfonyl]phenyl}acetamide
Metabolite501	Hydroxysuberic acid
Metabolite502	cis-5,8,11,14,17-Eicosapentaenoic acid
Metabolite503	(2R,3R,4S,5S,6R)-2-[2-(4-methoxyphenyl)ethoxy]-6-[[(2S,3R,4S,5S)-3,4,5-trihydroxyoxan-2-yl]oxymethyl]oxane-3,4,5-triol
Metabolite504	Aconitic Acid
Metabolite505	(2S,3R,4R,5R,6S)-2-[(2R,3R,4S,5R,6R)-2-[2-(3,4-dihydroxyphenyl)ethoxy]-3,5-dihydroxy-6-(hydroxymethyl)oxan-4-yl]oxy-6-methyloxane-3,4,5-triol
Metabolite506	3-[2-(β-D-Glucopyranosyloxy)-4-methoxyphenyl]propanoic acid
Metabolite507	Gentiopicrin
Metabolite508	{(1R,2R)-2-[(2Z)-5-(Hexopyranosyloxy)-2-penten-1-yl]-3-oxocyclopentyl}acetic acid
Metabolite509	Caffeic acid
Metabolite510	Harpagide
Metabolite511	LPE 16:0
Metabolite512	Robinin
Metabolite513	3-tert-Butyladipic acid
Metabolite514	5-[(3Z)-5-Hydroxy-3-methyl-3-penten-1-yl]-1,4a-dimethyl-6-methylenedecahydro-1-naphthalenecarboxylic acid
Metabolite515	Caffeic acid hexoside
Metabolite516	(+/-)9,10-dihydroxy-12Z-octadecenoic acid
